# Adenosine transmission from hypothalamic tanycytes to AGRP/NPY neurons regulates energy homeostasis

**DOI:** 10.1038/s12276-025-01449-6

**Published:** 2025-05-02

**Authors:** Nayoun Kim, Seolsong Kim, Seokjae Park, Eun-Kyoung Kim

**Affiliations:** 1 Department of Brain Sciences, Daegu Gyeongbuk Institute of Science and Technology, Daegu, Republic of Korea; 2 Neurometabolomics Research Center, Daegu Gyeongbuk Institute of Science and Technology, Daegu, Republic of Korea

**Keywords:** Hypothalamus, Obesity

## Abstract

Tanycytes are a pivotal component of the hypothalamic network that controls energy homeostasis. Despite their importance, the regulatory mechanisms governing tanycyte–neuron interactions in response to metabolic signals remain unexplored. Here we report that adenosine signaling between tanycytes and AGRP/NPY neurons is crucial for tanycytic metabolic regulation mediated by translocator protein 18 kDa (TSPO). Tanycyte-specific *Tspo*-knockout mice displayed reduced food consumption and weight loss associated with the downregulation of *Agrp* and *Npy* expression under high-fat diet feeding. *Tspo*-deficient tanycytes had elevated levels of intracellular ATP, which was released via connexin 43 hemichannels and extracellularly converted into adenosine by tanycytic ectonucleotidases. The adenosine signal was perceived by adenosine A1 receptors on adjacent AGRP/NPY neurons, reducing ERK phosphorylation, which in turn downregulated *Agrp* and *Npy* expression. Our findings underscore the anorexic role of adenosine as a gliotransmitter in the intricate communication between tanycytes and neurons for regulating appetite and body weight.

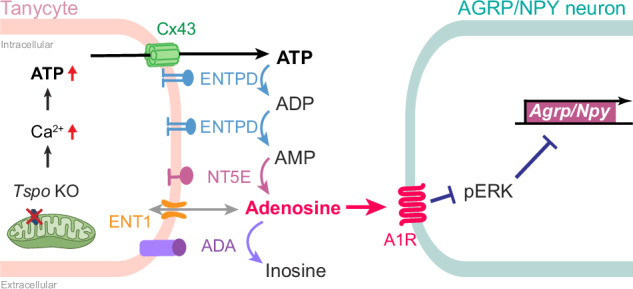

## Tanycytes regulate appetite through adenosine signaling pathway

Controlling body weight is a major health challenge worldwide, often due to an imbalance between energy intake and expenditure. The hypothalamus plays a key role in regulating hunger and energy use. This study explores how specific brain cells called tanycytes, which are in the hypothalamus, influence appetite and body weight. Researchers used mice with a genetic modification that removed TSPO from tanycytes to study its effects. These mice were fed a high-fat diet to mimic obesity conditions. The researchers found that removing TSPO led to reduced appetite, increased energy use and better glycemic control. This was linked to increased ATP release from tanycytes, which was then converted to adenosine that affected neurons controlling hunger. The study concludes that targeting tanycytes and their signaling pathways could offer new ways to treat metabolic disorders such as obesity.

This summary was initially drafted using artificial intelligence, then revised and fact-checked by the author.

## Introduction

Control of excess body weight is one of the greatest healthcare challenges worldwide. A chronic imbalance between energy intake and expenditure is the main etiology of obesity. The hypothalamus controls both hunger and systemic energy metabolism to maintain energy homeostasis by integrating metabolic signals such as nutrients or hormones^1,2^. Hypothalamic regulation of energy homeostasis in mammals is governed by the mediobasal hypothalamic neurons^1,2^. Besides that of neural circuits, the role of hypothalamic glial cells in controlling appetite and body weight is gaining increasing attention.

Hypothalamic tanycytes are highly specialized ependymoglial cells lining the third ventricle (3V) in the brain^3,4^. They are classified as α1, α2, β1 and β2 subtypes on the basis of their location^5^. Dorsal tanycytes located near the dorsomedial hypothalamus or ventromedial hypothalamus are referred to as α-tanycytes, while ventral tanycytes positioned near the arcuate nucleus (ARC) or median eminence (ME) are β-tanycytes^5^. Due to their shape and position, tanycytes sense nutrient signals, control the access of circulating hormones to the hypothalamus and shuttle metabolic factors that modulate the function of the neural circuits between blood or cerebrospinal fluid and hypothalamic neurons^4,6–13^. The cell bodies of tanycytes are exposed to the cerebrospinal fluid and send long extensions into the parenchyma of the dorsomedial hypothalamus, ventromedial hypothalamus, ARC and ME, connecting the central nervous compartments with peripheral information^3,8,14^. In particular, the tanycytic bridge to the ARC is critical for regulating energy homeostasis by integrating nutritional and hormonal signals delivered from the periphery through the ME, a circumventricular organ^3,4,8^. In the ARC, orexigenic neurons expressing agouti-related peptide (AGRP) and neuropeptide Y (NPY) stimulate food intake, whereas anorexigenic neurons expressing pro-opiomelanocortin (POMC) and cocaine- and amphetamine-regulated transcript (CART) promote satiety^15–17^. These neurons receive systemic messages about energy status by nutrients, hormones, neurotransmitters and gliotransmitters^18–22^.

Tanycytes have pivotal functions in energy metabolism, including the uptake of liraglutide for anti-obesity action, regulation of lipid metabolism through leptin entry, involvement in insulin uptake and mediation of inflammation-induced anorexia^10–12,23^. Tanycytes may deliver and transmit peripheral signals by releasing gliotransmitters, which play important roles in communication between tanycytes and adjacent neurons. It remains unclear which gliotransmitter(s) regulate tanycyte–neuron interactions and the mechanisms involved. It is, therefore, necessary for paradigm-shifting research on distinct tanycytic functions such as sensing, processing and transmitting blood-borne metabolic signals to adjacent neurons.

Mitochondria are the organelles highly relevant to metabolic signals. Translocator protein 18 kDa (TSPO), a protein located on the outer mitochondrial membrane, was known as the peripheral benzodiazepine receptor^24^. TSPO has been investigated in regulatory functions including steroidogenesis^25^, apoptosis^26^ and inflammation^27^. Although TSPO is highly expressed in glial cells in neuroinflammation-mediated neurodegeneration^28^, the roles of TSPO in the central nervous system were largely unknown. We have previously demonstrated the role of TSPO in tanycytes in the homeostatic responses to overnutrition^29^. Given the role attributed to tanycytes as mediators of energy homeostasis, the model of tanycytic *Tspo* deficiency is appropriate for investigating the communication between tanycytes and appetite-related neurons in regulating energy homeostasis. This study focused on unraveling the molecular entities and signaling pathways mediating communication between tanycytes and AGRP/NPY neurons using tanycyte-specific *Tspo*-knockout mice. Our results highlight the importance of tanycytes for therapeutic intervention in metabolic disorders.

## Materials and methods

### Animals

Rax-Cre^ERT2^ (*Rax*^*tm1.1(cre/ERT2)Sbls*^/J, JAX#025521), Ai14-tdTomato reporter (*ROSA26Sor*^*tm14(CAG-tdTomato)Hze*^/J, JAX#007914) and Tspo^loxp/loxp^ (*Tspo*^*tm1.1 Maf*^/J, JAX#024976) mice were purchased from The Jackson Laboratory. Rax-Cre^ERT2^ were bred with Ai14 reporter mice to generate Rax-Cre^ERT2^; Ai14 mice (Tan^*Tspo*^ wild type (WT)), which were bred with Tspo^f/f^ mice to generate tanycyte-specific *Tspo*-knockout mice expressing tdTomato reporter (Tan^*Tspo*^ cKO). To induce Cre recombination, male and female mice of both Tan^*Tspo*^ WT and Tan^*Tspo*^ cKO were administered double intraperitoneal (i.p.) injections of tamoxifen (100 mg/kg; Sigma-Aldrich, T5648) dissolved in corn oil (Sigma-Aldrich, C8267). NPY-hrGFP (JAX#006417), AgRP-ires-Cre (JAX#012899) and POMC-hrGFP (JAX#005765) mice were received from Dr. Ki-Woo Kim’s laboratory (Yonsei University). AgRP-ires-Cre mice were bred with Ai14-tdTomato reporter mice to generate AgRP^tdTom^ mice. Mice were housed under specific-pathogen-free conditions in a temperature-controlled room (20–26 °C) with 40–60% humidity under a 12 h light/dark cycle in individually ventilated cages. Mice were fed an ad libitum chow diet (LabDiet, #5053) containing 62% of calories from carbohydrates, 24% from protein and 13% from fat, or high-fat diet (HFD) (Envigo, TD.06414) containing 21% of calories from carbohydrates, 18% from protein and 60% from fat. All experiments and procedures used in this study were approved by the Institutional Animal Care and Use Committee at the DGIST Laboratory Animal Resource Center (approval number DGIST-IACUC-20042201-0001).

### Genotyping

Genomic DNA was extracted from mouse tail tips by using a lysis reagent (Fiat International, 101) with proteinase K (2 mg/ml; Sigma-Aldrich, P6556) at 55 °C overnight and inactivated proteinase K at 85 °C for 15 min. Genomic DNA was used for genotyping by PCR using the primer pairs and 2X Taq polymerase (SolGent, STD01-M50h) to determine Tspo^f/f^, Rax-Cre^ERT2^, Ai14-tdTomato and AgRP-ires-Cre alleles. The primer sequences are listed in Supplementary Table 1.

### Measurements of food intake and body weight

Food intake and body weight were measured daily at the start of the dark cycle in individualized male and female Tan^*Tspo*^ WT and Tan^*Tspo*^ cKO mice. After the intracerebroventricular (ICV) injection of ARL67156, DPCPX, adenosine or vehicle at the onset of the dark cycle, food intake and body weight were monitored at 3, 6, 12 and 24 h.

### Immunohistochemistry

Mice were anesthetized by i.p. injection of the mixture of Zoletil 50 (30 mg/kg; Virbac) and rompun (10 mg/kg; Bayer Korea), perfused intracardially with phosphate-buffered saline (pH 7.4) and fixed with 4% paraformaldehyde. Brain tissues were cryoprotected in 30% sucrose solution at 4 °C, frozen in optimal cutting temperature compound (Leica, 3801480) on dry ice, and stored at −80 °C. Tissues were cut into 30-µm sections on a cryostat (Leica), and the sections were blocked with 5% donkey serum (Jackson ImmunoResearch, 017-000-121), 1% bovine serum albumin (Sigma-Aldrich, A6003) and 0.3% Triton X-100 (Sigma-Aldrich, T8787) in phosphate-buffered saline for 2 h at room temperature. The sections were incubated with primary antibodies at 4 °C overnight and with fluorophore-conjugated secondary antibodies at room temperature for 2 h (Supplementary Table 2). The sections were then incubated with Hoechst 33342 (Thermo Scientific, 62249) for 5 min. For pERK staining, all solutions contained 50 mM NaF (Sigma-Aldrich, 201154) to inhibit phosphatase activity. Brain slices were mounted on glass slides (Muto Pure Chemicals Co., 5116-20 F), covered with cover glasses (Marienfeld, 0101222) using Vectashield antifade mounting medium (Vector Laboratories, H1000) and imaged on a Zeiss LSM 800 confocal microscope. Zen Blue 2.6 software (Carl Zeiss Microscopy) was used to acquire and analyze the original images. Manders’ overlap coefficients were used to quantify the colocalization of Rax with tdTomato (10 brain slices) or HA tag with tdTomato (12 brain slices) in Tan^*Tspo*^ WT mice. Colocalization coefficients were used to quantify the colocalization of A1R with GFP or A2bR with GFP in Npy-hrGFP (10 brain slices) and Pomc-hrGFP mice (8 to 10 brain slices). In AgRP^tdTom^;shScram or AgRP^tdTom^;sh*A1r* mice, the colocalization of A1R with EGFP or EGFP with tdTomato was quantified using 10 brain slices based on colocalization coefficients. To quantify pERK colocalized with EGFP, we analyzed 12 brain slices from AgRP^tdTom^;shScram or AgRP^tdTom^;sh*A1r* mice using Fiji in ImageJ 2 software (National Institutes of Health), as previously described^30^.

### Glucose and insulin tolerance tests

Glucose and insulin tolerance of Tan^*Tspo*^ WT and Tan^*Tspo*^ cKO mice were tested after 4 weeks of HFD feeding. Mice were starved for 16 h for glucose tolerance tests and 6 h for insulin tolerance tests. After i.p. injection of glucose (2 g/kg; Sigma-Aldrich, G7021) or human insulin (1 U/kg; Sigma-Aldrich, I9278), glucose was measured for 2 h in blood from the tail vein using an Accu-check II glucometer (Roche).

### Body composition analysis and indirect calorimetry

Tan^*Tspo*^ WT and Tan^*Tspo*^ cKO mice were anesthetized with isoflurane (Hana Pharm.) using anesthetic vaporizers (Harvard Apparatus), and body composition was measured by iNSiGHT DXA (Osteosys) after 4 weeks of HFD feeding. After these measurements, indirect calorimetry was performed using the Comprehensive Lab Animal Monitoring System (CLAMS, Columbus Instruments). Mice were housed individually with free access to water and HFD for 24 h to acclimatize, and indirect calorimetry data were measured during 24 h. VO_2_, VCO_2_, heat production and locomotor activities (*X* and *Z* axis) were determined, and the respiratory exchange ratio was automatically calculated as VCO_2_/VO_2_ using an Oxymax system (Columbus Instruments). FA oxidation was calculated using a previously described equation^31^, and energy expenditure was analyzed using analysis of covariance (ANCOVA) with body weight as a covariate, as previously described^32,33^.

### Generation of adeno-associated virus (AAV)

The viral vector pAAV[FLEXon]-CAG>LL:rev(NheI/HA/6xHis/thrombin_site:Luciferase:GPI/AscI):rev(LL):WPRE was constructed to obtain Cre-dependent expression of luciferase on the plasma membrane of tanycytes. The viral vectors pAAV[FLEXon]-CAG>LL:rev(EGFP:mAdora1[miR30-shRNA#1]):LL:WPRE and pAAV[FLEXon]-CAG>LL:rev(EGFP:Scramble[miR30-shRNA#1]):LL:WPRE, were constructed to suppress the genetic expression of adenosine A1 receptors in AGRP neurons and to generate the Cre-dependent control virus, respectively. AAV-PHP.eB[FLEXon]-CAG-LUC-GPI-HA, AAV-PHP.eB[FLEXon]-CAG-sh*A1r*-EGFP and AAV-PHP.eB[FLEXon]-CAG-shScram-EGFP were produced at VectorBuilder.

### AAV injection

For stereotaxic injection of AAV, all mice were anesthetized with the mixture of Zoletil 50 and rompun by i.p. injection. Tan^*Tspo*^ WT and Tan^*Tspo*^ cKO mice were injected with 2 μl of AAV-PHP.eB[FLEXon]-CAG-LUC-GPI-HA (1.22 × 10^13^ genome copies per milliliter) into the 3 V (1.3 mm posterior, 0.0 mm medial and 5.3 mm ventral to bregma) to generate Tan^*Tspo*^ WT-LUC and Tan^*Tspo*^ cKO-LUC mice, respectively. AgRP^tdTom^ mice were injected with 1.5 μl of AAV-PHP.eB[FLEXon]-CAG-sh*A1r*-EGFP (1.79 × 10^13^ genome copies per milliliter) or 1.5 μl of AAV-PHP.eB[FLEXon]-CAG-shScram-EGFP (1.90 × 10^13^ genome copies per milliliter) into the ARC (1.3 mm posterior, ±0.5 mm lateral and 5.6 mm ventral to bregma) to generate AgRP^tdTom^;sh*A1r* or AgRP^tdTom^;shScram mice, respectively. After the AAV injection, the scalp was sutured and mice were housed in individual cages for recovery for 2 weeks.

### Extracellular ATP bioluminescence imaging

Extracellular ATP bioluminescence imaging of the brain was performed starting at 15 min after i.p. injection of d-luciferin (150 mg/kg; GoldBio, 115144-35-9) in Tan^*Tspo*^ WT-LUC and Tan^*Tspo*^ cKO-LUC mice using an In Vivo Optical Imaging System (IVIS, PerkinElmer). Mice were anesthetized at each time point right before bioluminescence measurement with isoflurane using the XGI-8 Gas Anesthesia System during imaging (PerkinElmer). To perform ICV injection of Gap26 (0.25 nmol; Tocris, 1950)^34^ or vehicle into the 3V (1.3 mm posterior, 0.0 mm medial and 5.3 mm ventral to bregma), Tan^*Tspo*^ WT-LUC and Tan^*Tspo*^ cKO-LUC mice were anesthetized by i.p. injection of the mixture of Zoletil and rompun. The concentration of Gap26 for ICV injection was determined on the basis of the corresponding reference.

### Cannulation and ICV injection

For ICV injection, all mice were anesthetized by i.p. injection of the mixture of Zoletil 50 and rompun. Tan^*Tspo*^ WT and Tan^*Tspo*^ cKO mice were implanted with a unilateral cannula (PlasticsOne) at the 3V (1.3 mm posterior, 0.0 mm medial, and 5.3 mm ventral to bregma) to inject ARL67156 (0.1 nmol; Tocris, 1283)^35^ or vehicle or with a bilateral cannula (PlasticsOne) at the ARC (1.3 mm posterior, ±0.5 mm lateral and 5.6 mm ventral to bregma) to inject DPCPX (0.4 nmol; Tocris, 0439)^36^ or vehicle. WT mice were cannulated at the ARC (1.3 mm posterior, ±0.5 mm lateral and 5.6 mm ventral to bregma) to inject DPCPX (0.4 nmol), adenosine (10 nmol; Sigma-Aldrich, A9251)^37^ or vehicle. AgRP^tdTom^;shScram and AgRP^tdTom^;sh*A1r* mice were cannulated at the ARC (1.3 mm posterior, ±0.5 mm lateral and 5.6 mm ventral to bregma) to inject adenosine (10 nmol) or vehicle. The vehicle for DPCPX was 4% ethanol diluted in saline; all other vehicles for ICV injection were saline. After the cannula implantation, the scalp was covered with dental cement (Dentsply, LAD010) and mice were housed in individual cages for recovery for 10 days. The concentration of the drugs used for icv injection was determined on the basis of the corresponding references.

### Cell culture

The mHypoA-A2/29 (CLU189), mHypoE-N41 (CLU121) and mHypoE-N43/5 (CLU127) cell lines were purchased from Cellutions Biosystems and maintained in DMEM culture medium (Sigma-Aldrich, D5796) supplemented with heat-inactivated (at 55 °C) 10% fetal bovine serum (Hyclone Laboratories, SH30910.03) and 1% penicillin–streptomycin (Hyclone Laboratories, SH30236.01). Cell lines were tested for mycoplasma by Cellutions Biosystems (no authentication).

### Indirect coculture system

Cells were cultured in a transwell system using 0.4-μm pore membrane inserts (Corning, 3450). A2/29 cells were grown on permeable membranes for 24 h, and N41 or N43/5 cells were grown separately in the lower well for 24 h. These cells were transfected with small interfering RNAs (siRNAs). The medium was changed to fresh medium 4 h after transfection, and the insert wells were placed over the lower wells that are N41 or N43/5 cells to share the medium for 44 h.

### Drug treatments

A2/29 cells were treated with Gap26 (0.05 mM), ARL67156 (0.1 mM), APCP (0.1 mM; Tocris, 3633), EHNA (0.01 mM; Tocris, 1261), NBMPR (0.01 mM; Tocris, 0625) or vehicle. N41 cells were treated with adenosine (0.1 mM), EHNA (0.01 mM) or DPCPX (0.01 mM). N43/5 cells were treated with adenosine (0.1 mM) and EHNA (0.01 mM). In the indirect coculture system, oleic acid (0.06 mM; Sigma-Aldrich, O7501) was added to shared media supplemented with ARL67156 (0.1 mM), APCP (0.1 mM), EHNA (0.01 mM), NBMPR (0.01 mM) or vehicle for 3 h before the media were collected and the cells collected for analyses of ATP and adenosine levels, or mRNA expression. The vehicles for Gap26, ARL67156, APCP and EHNA were sterilized water. The vehicle for NBMPR was dimethyl sulfoxide (Sigma-Aldrich, D2650), and that for DPCPX was ethanol. All concentrations of drugs used in cell lines were within the range that did not reduce cell viability.

### Transfection of siRNA

Cells were transfected with *Tspo*, *A1r* or *A2br* siRNA (100 nM) using Lipofectamine 3000 (Life Technologies, L3000-015) according to the manufacturer’s instructions. *Tspo* siRNA (L-040291-02-0005), *A1r* siRNA (L-063568-00-0005) and *A2br* siRNA (L-060263-00-0005) were SMARTpools produced by Dharmacon. Scrambled siRNA (100 nM; ON-TARGETplus Non-targeting Pool, D-001810-10-20) from Dharmacon was used as a transfection control.

### ATP assay

Intracellular and extracellular ATP levels were measured by the ATP Bioluminescence Assay Kit HS II (Roche, 11-699-709-001) according to the manufacturer’s instructions. For measuring extracellular ATP levels, medium was collected. For measuring intracellular ATP levels, cells were lysed with TE buffer, heated at 95 °C for 7 min and centrifuged (21,130*g*, 4 °C) for 3 min. Collected extracellular media and cell lysates were mixed with luciferase reagent, and luminescence was detected using a luminescence reader (SpectraMax-L microplate reader, Molecular Devices). The ATP levels were normalized to the amount of total protein quantified using a bicinchoninic acid (BCA) assay kit (Thermo Fisher Scientific, 23225) in cell lysates.

### Adenosine assay

Intracellular and extracellular adenosine levels were measured by using an adenosine assay kit (BioVision, K327-100) according to the manufacturer’s protocol. Medium was collected, and cells were lysed and centrifuged as for the ATP assay. Collected extracellular media and cell lysates were mixed with detection reagents, and fluorescence intensity was measured at excitation/emission 535/587 nm using a fluorescence spectrophotometer (SpectraMax Gemini EM microplate reader, Molecular Devices). The adenosine levels were normalized to the amount of total protein quantified using a BCA assay kit in cell lysates.

### Real-time qPCR

Samples were prepared as previously described^29^. Real-time qPCR was performed in a qPCR machine (CFX96 Real-Time PCR, Bio-Rad). The primers were synthesized by Bionics (Supplementary Table 3). The reaction mixture consisted of 3 μl of cDNA template (1 μg/μl), 10 μl of TB Green PCR master mix (TaKaRa Biotechnology, RR820L) and 5 pmol of each gene-specific primer in a total volume of 20 μl. The cDNA was denatured at 95 °C for 1 min, followed by 40 cycles of PCR at 95 °C for 10 s, and 60 °C for 30 s. We used the ∆∆Cq method to analyze the relative expression levels of genes and that of *Gapdh* control. The qPCR experiments included three sets of biological replicates, with each set consisting of an average of three technical replicates.

### Western blot

Cells were lysed as previously reported^30^. Concentrations of the lysed proteins were measured with a BCA assay kit using a VersaMax microplate reader (Molecular Devices). The equivalent amounts of protein (9 μg) were separated on SDS–PAGE gels and transferred to Immobilon-P membrane (Merck, IPVH00010). Membranes were blocked by 5% skim milk in TBST (20 mM Tris, 125 mM NaCl and 0.1% Tween 20, pH 7.4) for 1 h. The membranes were incubated with the primary antibody for 2 h at room temperature or overnight at 4 °C. Then, the membranes were incubated with horseradish-peroxide-conjugated secondary antibody for 1 h at room temperature. The membranes were exposed to the SuperSignal West Pico Chemiluminescent Substrate (Thermo Fisher Scientific, 34580), and signal intensity was quantified using ImageJ software (National Institutes of Health). The antibodies are listed in Supplementary Table 2.

### Statistical analysis

Data were presented as mean ± s.e.m. or boxes indicating the interquartile range with whiskers extending from the box to the highest and lowest values, or truncated violin plots. All statistical analyses were performed using GraphPad Prism 9.1.1 (GraphPad Software). Significance was determined by unpaired two-tailed Student’s *t*-test to compare two groups or one-way analysis of variance (ANOVA) and two-way ANOVA to compare several groups. Differences in energy expenditure were calculated by ANCOVA using body weight as a covariate. *P* values of less than 0.05 were considered statistically significant.

## Results

### Genetic deletion of TSPO in tanycytes reduces appetite, elevates energy expenditure and improves glucose homeostasis in diet-induced obese (DIO) mice

**Fig. 1:**
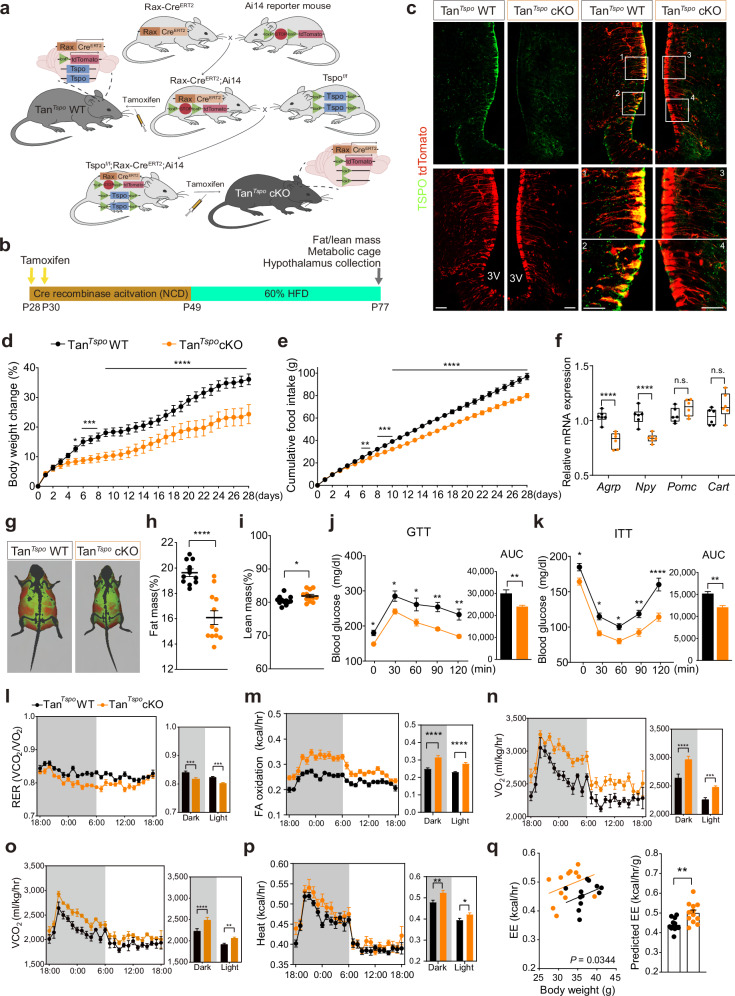
Genetic deletion of TSPO in tanycytes reduces appetite, elevates energy expenditure and improves glucose homeostasis in DIO mice. **a** Rax-Cre^ERT2^ mice bred with Ai14-tdTomato reporter mice to generate control (Tan^*Tspo*^ WT) mice. Rax-Cre^ERT2^; Ai14 mice bred with Tspo^f/f^ mice to generate tanycyte-specific Tan^*Tspo*^ cKO mice. **b** A schematic diagram showing the timing for i.p. administration of tamoxifen and shift from normal chow diet (NCD) to HFD. Tan^*Tspo*^ WT and Tan^*Tspo*^ cKO mice were i.p. injected with tamoxifen at P28 and P30 to activate Cre recombinase. Three weeks after tamoxifen administration, mice were fed HFD for 4 weeks. **c** Representative images of immunostaining for TSPO (green) in tanycytes labeled with tdTomato (red). **d**, **e** Body weight change (**d**) and cumulative food intake (**e**) during 4 weeks of HFD in Tan^*Tspo*^ WT and Tan^*Tspo*^ cKO mice (*n* = 12 per group). **f** Relative mRNA levels of neuropeptides in the mouse hypothalamus at P77 (*n* = 6 per group). **g** Representative images of body composition. Red, fat mass; green, lean mass. **h**, **i** Fat mass (**h**) and lean mass (**i**) at P77 (*n* = 12 per group). **j**, **k** Glucose tolerance test (GTT; *n* = 8 per group) (**j**) and insulin tolerance test (ITT; *n* = 8 per group) (**k**) in Tan^*Tspo*^ WT and Tan^*Tspo*^ cKO mice. **l**–**q** Indirect calorimetry parameters in Tan^*Tspo*^ WT and Tan^*Tspo*^ cKO mice (*n* = 12 per group): respiratory exchange ratio (RER) (**l**) fatty acid (FA) oxidation (**m**) O_2_ consumption (VO_2_) (**n**) CO_2_ production (VCO_2_) (**o**), heat generation (**p**) and regression plots of energy expenditure (EE) against body weight and predicted EE at the mean body weight of each group (**q**). Scale bar, 20 μm (**c**). Data are mean ± s.e.m. or boxes indicating the interquartile range with whiskers. Significance was determined by two-tailed unpaired Student’s *t*-test (*P < 0.05, **P < 0.01, ****P < 0.0001) in **h** and **i** AUC in **j** and **k** and ANCOVA in **q** or otherwise by two-way ANOVA with Šidák’s multiple-comparisons test (*P < 0.05, **P < 0.01, ***P < 0.001, ****P < 0.0001). n.s., not significant; AUC, area under the curve.

To investigate the mechanisms underlying the interaction between tanycytes and ARC neurons related to anorexia and anti-obesity effects, we generated tanycyte-specific *Tspo* conditional knockout (Tan^*Tspo*^ cKO) male mice (Fig. 1a). Three weeks after the first i.p. tamoxifen injection at P28, mice were fed a HFD for 4 weeks (Fig. 1b). We chose P28 for tamoxifen injection as it was previously reported that Cre recombinase activity in Rax-Cre^ERT2^ mice is more efficient at P28 than P50^38^. The expression of TSPO was markedly reduced in tanycytes but not in the mediobasal hypothalamus of Tan^*Tspo*^ cKO mice compared with Tan^*Tspo*^ WT mice (Fig. 1c and Supplementary Fig. 1a, b). At the end of HFD feeding, the body weight increase was lower in Tan^*Tspo*^ cKO mice than in Tan^*Tspo*^ WT mice by approximately 12% (Fig. 1d and Supplementary Fig. 1c). The cumulative food intake was lower in Tan^*Tspo*^ cKO mice than in Tan^*Tspo*^ WT mice by approximately 17 g (Fig. 1e and Supplementary Fig. 1d). However, metabolic parameters in Tan^*Tspo*^ cKO mice were comparable to those in Tan^*Tspo*^ WT mice under normal chow diet feeding for 4 weeks (Supplementary Fig. 1e, f). Based on these results, we examined whether TSPO in tanycytes regulated food intake and body weight by modulating the expression of orexigenic or anorexigenic neuropeptides under HFD feeding. Relative mRNA expression of *Agrp* and *Npy* was significantly lower in Tan^*Tspo*^ cKO mice than in Tan^*Tspo*^ WT mice, but no differences were found for *Pomc* and *Cart* mRNA (Fig. 1f). Body composition analysis indicated that the fat mass of Tan^*Tspo*^ cKO mice was significantly lower than that of Tan^*Tspo*^ WT mice, with a slight difference in lean mass (Fig. 1g–i). Tan^*Tspo*^ cKO mice had improved glucose tolerance and insulin sensitivity, along with lower fasting blood glucose levels compared with Tan^*Tspo*^ WT mice (Fig. 1j, k). The respiratory exchange ratio of Tan^*Tspo*^ cKO mice was lower, and fatty acid oxidation was higher than those of Tan^*Tspo*^ WT mice, indicating that Tan^*Tspo*^ cKO mice utilized more fat as a fuel source (Fig. 1l, m). Oxygen consumption, carbon dioxide production, heat production and energy expenditure were higher in Tan^*Tspo*^ cKO mice than in Tan^*Tspo*^ WT mice, with no differences in locomotor activities in both dark and light cycles (Fig. 1n–q and Supplementary Fig. 1g, h). During the indirect calorimetry measurements, food intake was consistently lower in Tan^*Tspo*^ cKO mice compared with Tan^*Tspo*^ WT mice (Supplementary Fig. 1i).

To investigate the gender differences, we also determined the effect of Tan^*Tspo*^ cKO on body weight, food intake, the mRNA expression of appetite-related neuropeptides, fat and lean mass, glucose tolerance and insulin sensitivity, and the indirect calorimetry parameters in females (Supplementary Fig. 2). Energy expenditure of Tan^*Tspo*^ cKO females showed no statistical difference due to a larger deviation of body weight than males (Supplementary Fig. 2r). Our data demonstrated that Tan^*Tspo*^ cKO elicits comparable effects regardless of gender under HFD feeding. Therefore, all subsequent experiments were consistently performed in Tan^*Tspo*^ cKO and Tan^*Tspo*^ WT male mice.

### Genetic deletion of TSPO in tanycytes increases ATP production and subsequent release through connexin 43 hemichannels

**Fig. 2:**
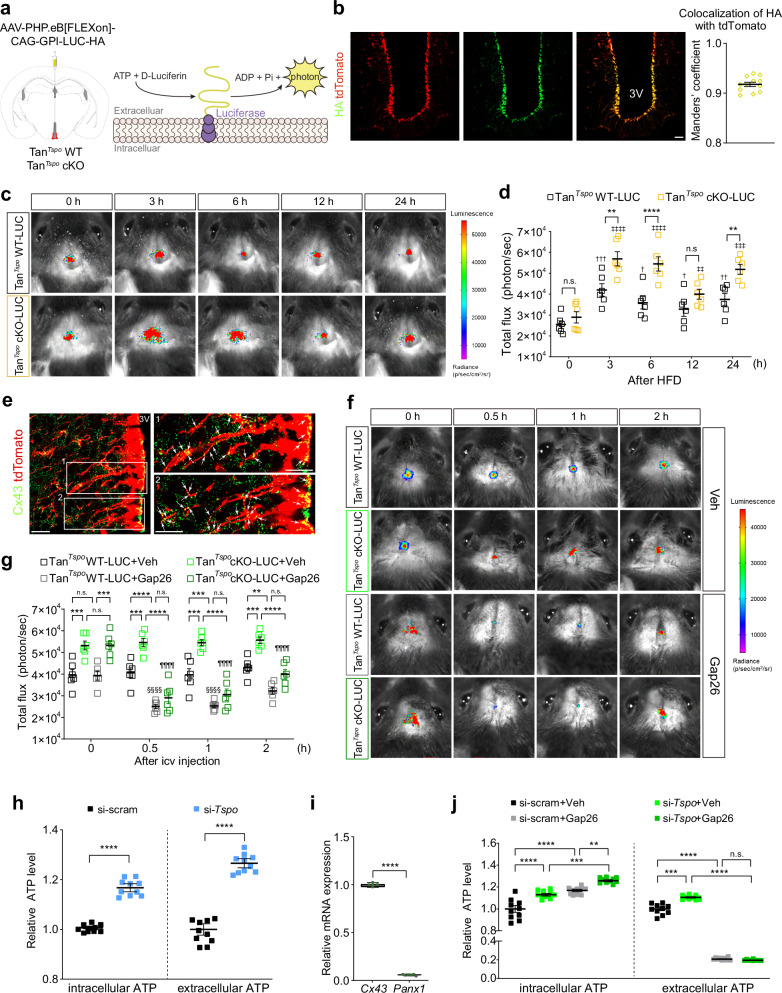
Genetic deletion of TSPO in tanycytes induces ATP production and release through connexin 43 hemichannels. **a** Left: a schematic illustration of a specific expression of luciferase on the plasma membrane of tanycytes in Tan^*Tspo*^ WT or Tan^*Tspo*^ cKO mice. Right: in the presence of ATP in the extracellular space, luciferase catalyzes the conversion of d-luciferin to emit a photon. **b** Representative immunohistochemistry images showing expressed HA tag (green) in tanycytes (red) and quantification graph of colocalization using Manders’ coefficient of HA and tdTomato (*n* = 12). **c**, **d** Representative photographs of bioluminescence imaging (**c**) and quantification of total flux expressed as photon per second (**d**) in Tan^*Tspo*^ WT-LUC and Tan^*Tspo*^ cKO-LUC mice during 24 h of HFD feeding (*n* = 6 per group). **e** Representative immunostaining images of connexin 43 hemichannels (Cx43; green) on tanycytes (red). The white box in the left image is enlarged in the right image, where arrowheads indicate Cx43. **f**, **g** Representative bioluminescence images (**f**) and quantification of total flux (**g**) after vehicle (Veh) or 0.25 nmol Gap26 (Cx43 blocker) ICV injection into the 3V of Tan^*Tspo*^ WT-LUC and Tan^*Tspo*^ cKO-LUC mice after 24 h of HFD feeding (*n* = 6 per group). **h** Relative intracellular and extracellular ATP levels in control (si-scram) or *Tspo*-knockdown (si-*Tspo*) tanycyte-like cells (mHypoA-A2/29, A2/29) (*n* = 9 per group). **i** Relative mRNA levels of *Cx43* and *Pannexin-1* (*Panx1*) in A2/29 cells (*n* = 6). **j** Relative intracellular and extracellular levels of ATP in si-scram or si-*Tspo* A2/29 cells in the presence or absence of 0.05 mM Gap26 for 0.25 h (*n* = 9 per group). Scale bars, 20 μm (**b** and **e**). Data are mean ± s.e.m. or boxes indicating the interquartile range with whiskers, and dotted lines indicate that significance was calculated separately for each. Significance was determined by two-tailed unpaired Student’s *t*-test (****P < 0.0001) in **h** and **i** or otherwise by two-way ANOVA with Šidák’s multiple-comparisons test: **P < 0.01, ***P < 0.001, ****P < 0.0001; ^†^*P* < 0.05, ^††^*P* < 0.01, ^†††^*P* < 0.001 versus Tan^*Tspo*^ WT^-^LUC 0 h; ^‡‡^*P* < 0.01, ^‡‡‡^*P* < 0.001; ^‡‡‡‡^*P* < 0.0001 versus Tan^*Tspo*^ cKO-LUC 0 h; ^§§§§^*P* < 0.0001 versus Tan^*Tspo*^ WT-LUC+Gap26 0 h; ^¶¶¶¶^*P* < 0.0001 versus Tan^*Tspo*^ cKO-LUC+Gap26 0 h. n.s., not significant.

Our previous study showed that the knockdown of *Tspo* in tanycyte-like cells in vitro promotes intracellular ATP production under oleic acid treatment^29^, which mimics the lipid-rich cellular environment observed in mice fed HFD. Therefore, we aimed to investigate whether tanycytic *Tspo* deficiency increases ATP release in HFD-fed mice. To monitor local ATP release in the hypothalamus in real time, we designed an AAV construct that allows the expression of chimeric luciferase-based protein biosensors anchored in cell membranes in a Cre-dependent manner, as shown previously^39,40^. ICV injection of AAV-PHP.eB-[FLEXon]-CAG-GPI-LUC-HA into the 3V in Tan^*Tspo*^ WT and Tan^*Tspo*^ cKO mice fed normal chow diet was performed to generate Tan^*Tspo*^ WT-LUC mice and Tan^*Tspo*^ cKO-LUC mice, respectively, which express luciferase on the surface of tanycytes (Fig. 2a). The presence of luciferase in tanycytes was demonstrated via the expression of HA tag (luciferase) colocalized with tdTomato (tanycytes) 2 weeks after ICV injection (Fig. 2b).

To assess the extracellular ATP levels in tanycytes, we recorded bioluminescence in Tan^*Tspo*^ WT-LUC and Tan^*Tspo*^ cKO-LUC mice i.p. injected with d-luciferin and fed HFD for 24 h. HFD feeding was initiated at the onset of the dark cycle to coincide with the period of increased feeding activity. The bioluminescence was significantly higher in Tan^*Tspo*^ cKO-LUC than in Tan^*Tspo*^ WT-LUC mice at 3, 6 and 24 h but not at 12 h after HFD feeding (Fig. 2c, d). The absence of a significant difference at 12 h might be attributed to the beginning of the light cycle, during which food consumption tends to be lower than during the dark cycle. When HFD feeding was extended, bioluminescence was still higher at 48 and 72 h in Tan^*Tspo*^ cKO-LUC mice than in Tan^*Tspo*^ WT-LUC mice (Supplementary Fig. 3a, b). As these observations indicated high levels of extracellular ATP in Tan^*Tspo*^ cKO-LUC mice, we investigated the mechanisms by which ATP was released from tanycytes. To determine the contribution of connexin 43 (Cx43) hemichannels in vivo, we analyzed the expression of Cx43 by immunohistochemistry. Cx43 hemichannels were expressed at the edge of cell bodies of tanycytes along the wall of the 3V and tanycytic processes extending into the parenchyma (Fig. 2e). Next, we assessed ATP release after ICV injection of Gap26, a Cx43 hemichannel blocker, into the 3V. Gap26 efficiently dampened bioluminescence in both Tan^*Tspo*^ cKO-LUC and Tan^*Tspo*^ WT-LUC mice; in particular, it significantly decreased the elevated bioluminescence in Tan^*Tspo*^ cKO-LUC mice to levels similar to those in Tan^*Tspo*^ WT-LUC mice (Fig. 2f, g). These results indicate that ATP elevated by tanycytic *Tspo* deletion is released through Cx43 hemichannels.

To elucidate the mechanisms underlying the ATP release by tanycytic TSPO deletion, we used a hypothalamic cell line, mHypoA-A2/29 cell line (hereafter referred to as A2/29 cells) which is generated from the hypothalamus of an adult male mouse. As our previous study demonstrated^29^, A2/29 cells express vimentin and nestin, tanycyte markers, as well as *Rax* and *Tspo*, which are abundantly expressed in tanycytes (Supplementary Fig. 3c, d), making this a suitable model to study tanycytic TSPO-mediated functions. Therefore, the robust expression of *Rax* with other markers indicates that A2/29 displays tanycytic characteristics. To confirm that ATP elevated by *Tspo* knockdown was released from tanycytes, we assessed the intracellular and extracellular ATP levels in A2/29 cells. The levels of intracellular and extracellular ATP were higher in *Tspo*-knockdown A2/29 cells than in the scrambled control (Fig. 2h and Supplementary Fig. 3e). Previous studies suggested ATP release via Cx43 hemichannels or pannexin-1 (Panx1) in tanycytes^41,42^. Relative mRNA abundance of *Cx43* was much higher than that of *Panx1* in A2/29 cells and the whole hypothalamus (Fig. 2i and Supplementary Fig. 3f). To clarify whether ATP was released via Cx43 hemichannels in *Tspo*-knockdown A2/29 cells, we blocked Cx43 hemichannels with Gap26, which effectively blocked ATP release and led to higher intracellular ATP levels in *Tspo*-knockdown cells than the scrambled control (Fig. 2j). Consequently, our data suggest that ATP is released via Cx43 hemichannels in *Tspo*-knockdown tanycytes.

### ATP released from *Tspo*-knockdown tanycytes is converted to adenosine extracellularly by tanycytic ENTPD1 and NT5E

**Fig. 3:**
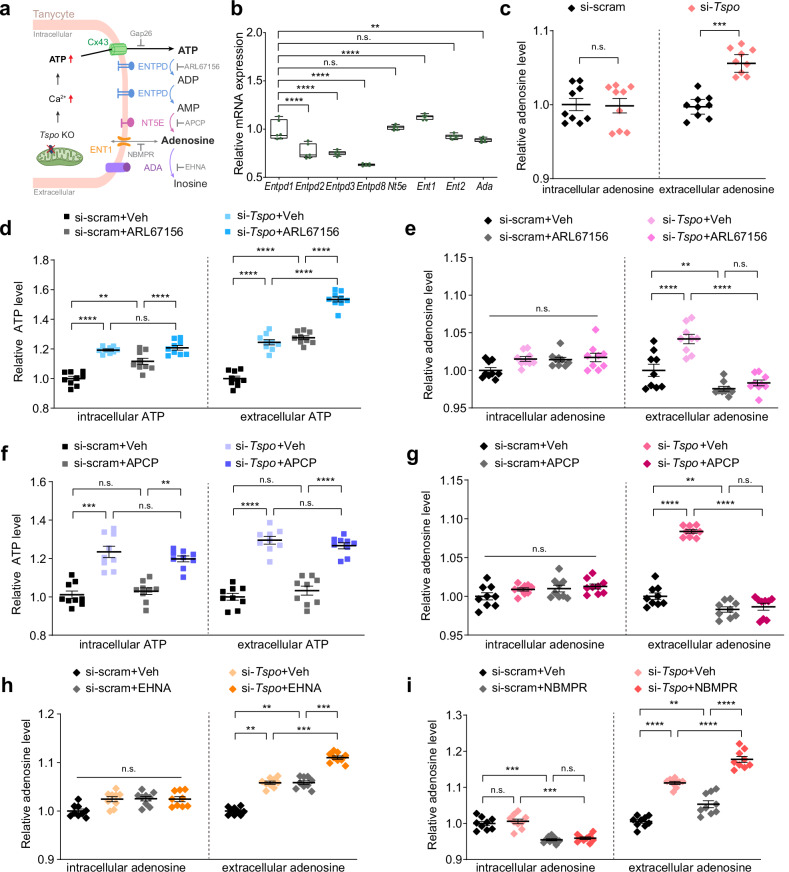
ATP released from *Tspo*-knockdown tanycytes is converted to adenosine extracellularly by tanycytic ENTPD and NT5E. **a** A schematic diagram of ATP release from tanycytes and conversion of nucleotides by ectonucleotidases. ATP is released through Cx43 from tanycytes and is converted to ADP and AMP by ENTPD. AMP is converted to adenosine by NT5E. Adenosine is transported by ENT1 and is converted to inosine by ADA. **b** Relative expression levels of ectonucleotidases families and nucleoside transporters in A2/29 cells (*n* = 6). **c** Relative intracellular and extracellular adenosine levels in si-scram and si-*Tspo* A2/29 cells (*n* = 9 per group). **d**, **f** Relative intracellular and extracellular ATP levels in si-scram and si-*Tspo* A2/29 cells in the presence or absence of ENTPD inhibitor (ARL67156, 0.1 mM) (**d**) or NT5E inhibitor (APCP, 0.1 mM) (**f**) for 3 h (*n* = 9 per group). **e,** **g**–**i** Relative intracellular and extracellular adenosine levels in si-scram and si-*Tspo* A2/29 cells in the presence or absence of ARL67156 (**e**) APCP (**g**) ADA inhibitor (EHNA, 0.01 mM) (**h**) or ENT1 inhibitor (NBMPR, 0.01 mM) (**i**) for 3 h (*n* = 9 per group). Data are mean ± s.e.m. or boxes indicating the interquartile range with whiskers, and dotted lines indicate that significance was calculated separately for each. Significance was determined by one-way ANOVA with Dunnett’s multiple-comparisons test (**P < 0.01, ****P < 0.0001) in **b** two-tailed unpaired Student’s *t*-test (***P < 0.001) in **c** or otherwise by two-way ANOVA with Šidák’s multiple-comparisons test (**P < 0.01, ***P < 0.001, ****P < 0.0001). n.s., not significant.

Extracellular ATP is rapidly converted to adenosine^43,44^. Therefore, our next question was whether ATP released from *Tspo-*knockdown tanycytes is converted to adenosine. ATP is extracellularly converted to adenosine via ectonucleotidases, such as ectonucleoside triphosphate diphosphohydrolase (ENTPD), ecto-5′-nucleotidase (NT5E) and adenosine deaminase (ADA)^45^. Extracellular adenosine is transported into the cell via an equilibrative nucleoside transporter (ENT) (Fig. 3a)^45^. The families of ectonucleotidases and transporters were present in A2/29 cells; in particular, subtype 1 of ENTPD and ENT, NT5E and ADA were dominant (Fig. 3b). The high mRNA levels of *Entpd1* and *N*t5e in the mouse hypothalamus (Supplementary Fig. 3g) and the expression of ENTPD1 and NT5E proteins in tanycytes (Supplementary Fig. 3h, i) were confirmed.

Given that *Tspo* knockdown increased the release of ATP into the extracellular medium, we expected an increase in the extracellular levels of adenosine. Indeed, they were significantly elevated upon *Tspo* knockdown in A2/29 cells, while intracellular adenosine levels were not changed (Fig. 3c).

We used pharmacological inhibitors of ATP-converting enzymes to interfere with the conversion of ATP to adenosine in A2/29 cells. ARL67156, a global ENTPD inhibitor, increased the levels of extracellular ATP and decreased those of extracellular adenosine regardless of *Tspo* knockdown, leading to extracellular ATP accumulation (Fig. 3d, e). Adenosine 5′-(α,β-methylene) diphosphate (APCP), an NT5E inhibitor, blunted the elevation of extracellular adenosine levels by *Tspo* knockdown but did not affect extracellular ATP levels, indicating the effective conversion of ATP to AMP (Fig. 3f, g). EHNA, an ADA inhibitor, further elevated the extracellular adenosine levels in *Tspo*-knockdown cells (Fig. 3h). These data suggest that *Tspo* knockdown does increase extracellular adenosine levels, despite the conversion of extracellular adenosine to inosine by ADA.

NBMPR, an ENT1 inhibitor, decreased intracellular adenosine levels regardless of *Tspo* knockdown, indicating that NBMPR blocked the transport of extracellular adenosine into the cells. By contrast, extracellular adenosine levels were further increased by NBMPR in *Tspo*-knockdown cells (Fig. 3i). These results show that substantial amounts of extracellular adenosine were converted from ATP despite the extracellular adenosine entering the tanycytes through ENT1.

Despite the conversion of extracellular adenosine to inosine by ADA (Fig. 3h) and its uptake into cells by ENT1 (Fig. 3i), extracellular adenosine levels were increased considerably in *Tspo*-knockdown cells (Fig. 3c). Rapid metabolism of ATP to adenosine probably overcame the conversion of adenosine to inosine and the influx of the extracellular adenosine. Taken together, these data indicate that ATP released by *Tspo*-knockdown cells was converted to adenosine, which is present extracellularly in tanycytes.

### Adenosine extracellularly converted from ATP downregulates the expression of *Agrp* and *Npy*

To investigate the communication between tanycytes and orexigenic neurons in regulating the expression of *Agrp* and *Npy*, we cocultured A2/29 cells and AGRP/NPY-expressing neuronal cell line (mHypoE-N41, hereafter referred to as N41) (Supplementary Fig. 4a). The coculture was exposed to the medium containing oleic acid to mimic the HFD condition in vitro. Extracellular ATP and adenosine levels in *Tspo*-knockdown cells were increased by oleic acid (Supplementary Fig. 4b, c).

**Fig. 4:**
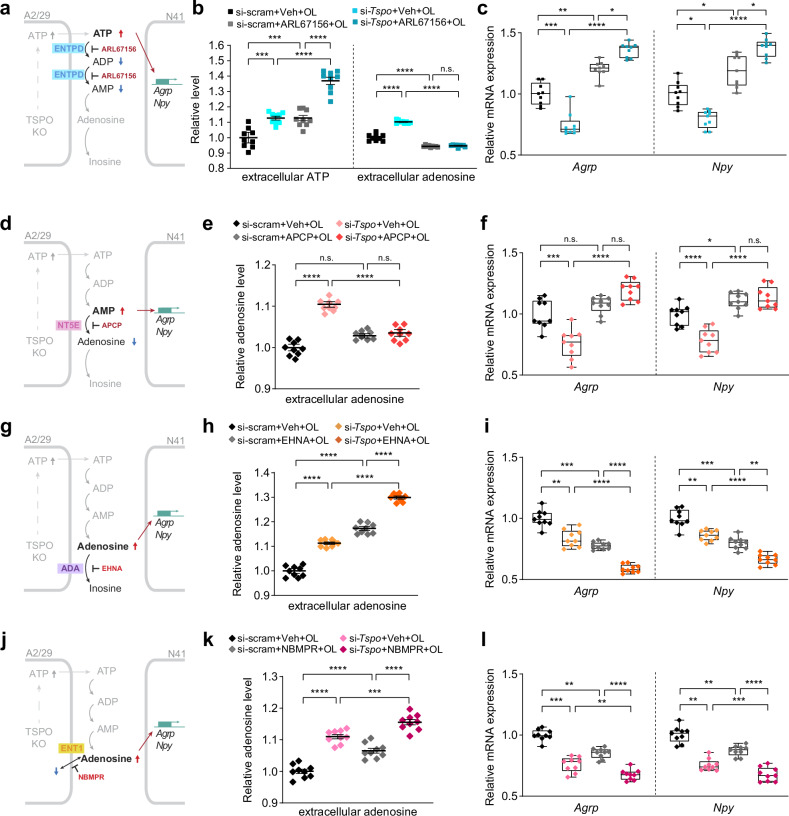
Adenosine converted from extracellular ATP downregulates the expression of *Agrp* and *Npy.* **a**, **d**, **g**, **j** Schematic illustrations of ARL67156 (**a**) APCP (**d**) EHNA (**g**) and NBMPR (**j**) treatments in the indirect coculture system. **b**, **e**, **h**, **k** Relative extracellular ATP or adenosine levels in si-scram or si-*Tspo* A2/29 cells indirectly cocultured with N41 cells in the presence or absence of ARL67156 (**b**) APCP (**e**) EHNA (**h**) or NBMPR (**k**) under 0.06 mM oleic acid (OL) treatment for 3 h (*n* = 9 per group). **c**, **f**, **i**, **l** Relative mRNA levels of *Agrp* and *Npy* in N41 cells cocultured with si-scram or si-*Tspo* A2/29 cells in the presence or absence of ARL67156 (**c**), APCP (**f**) EHNA (**i**) or NBMPR (**l**) under OL treatment for 3 h (*n* = 9 per group). Data are mean ± s.e.m. or boxes indicating the interquartile range with whiskers, and dotted linesindicate that significance was calculated separately for each. Significance was determined by two-way ANOVA with Šidák’s multiple-comparisons test (*P < 0.5, **P < 0.01, ***P < 0.001, ****P < 0.0001; n.s., not significant).

ARL67156 was used to inhibit the conversion of ATP released from A2/29 cells (Fig. 4a). The mRNA analysis in the coculture system showed that *Tspo* knockdown in A2/29 cells significantly decreased the *Agrp* and *Npy* mRNA expression in N41 cells (Fig. 4c). Of interest, the downregulation of *Agrp* and *Npy* was completely reversed by ARL67156. As extracellular ATP and adenosine increased by *Tspo* knockdown and oleic acid were oppositely changed by ARL67156 with further increase in ATP and decrease in adenosine (Fig. 4b), these data suggest that further accumulation of extracellular ATP caused by the inhibition of its conversion upregulated the expression of *Agrp* and *Npy*; by contrast, adenosine converted from ATP downregulated their expression. Therefore, we hypothesized that the downregulation of *Agrp* and *Npy* expression by *Tspo* knockdown and oleic acid was due to adenosine converted from released ATP.

To further test whether extracellular adenosine downregulates the expression of *Agrp* and *Npy*, we selectively decreased the concentration of extracellular adenosine with preservation of ATP levels using APCP (Fig. 4d). In *Tspo*-knockdown A2/29 cells, APCP effectively suppressed the elevation of extracellular adenosine, but not ATP (Fig. 4e and Supplementary Fig. 4d). Under this condition, *Agrp* and *Npy* expression was not downregulated (Fig. 4f). Also, the extracellular adenosine levels were elevated by EHNA, resulting in a significant decrease in the expression levels of *Agrp* and *Npy* (Fig. 4g–i). NBMPR further increased extracellular adenosine levels in oleic-acid-treated *Tspo*-knockdown cells. In line with the inhibition of adenosine-to-inosine conversion, the expression levels of *Agrp* and *Npy* were decreased (Fig. 4j–l). In both experiments, the degree of *Agrp* and *Npy* downregulation was proportional to the elevated extracellular adenosine levels, supporting our hypothesis that extracellular adenosine downregulates the expression of *Agrp* and *Npy*, while ATP plays the opposite role. These findings indicate that conversion of extracellular ATP released from tanycytes to adenosine downregulates the expression of orexigenic neuropeptides in the hypothalamus.

### Adenosine reduces *Agrp* and *Npy* expression via adenosine A1 receptors

**Fig. 5:**
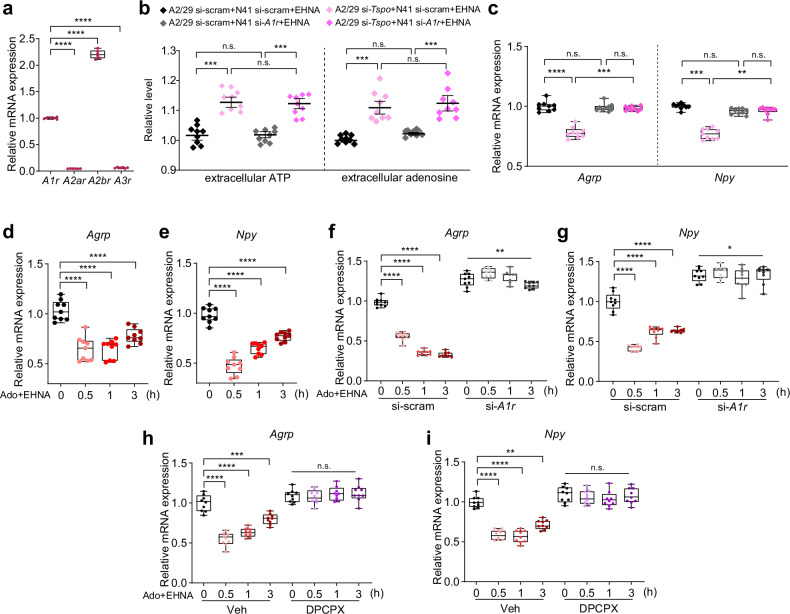
Adenosine reduces *Agrp* and *Npy* neuropeptide expression via adenosine A1 receptors. **a** Relative expression levels of adenosine receptors in A2/29 cells (*n* = 6). **b** Extracellular ATP levels and extracellular adenosine levels in si-scram or si-*Tspo* A2/29 cells indirectly cocultured with si-scram or si-*A1r* N41 cells for 3 h in the presence of EHNA (*n* = 9 per group). **c** Relative *Agrp* and *Npy* expression levels in si-scram or si-*A1r* N41 cells indirectly cocultured with si-scram or si-*Tspo* A2/29 cells in the presence of EHNA for 3 h (*n* = 9 per group). **d**, **e** Time courses of relative *Agrp* (**d**) and *Npy* (**e**) expression levels in N41 cells cotreated with 0.1 mM adenosine and 0.01 mM EHNA (Ado+EHNA) (*n* = 9 per group). **f**, **g** Time courses of relative *Agrp* (**f**) and *Npy* (**g**) expression levels in si-scram or si-*A1r* N41 cells treated with Ado+EHNA (*n* = 9 per group). **h**, **i** Relative *Agrp* (**h**) and *Npy* (**i**) expression levels in N41 cells treated with Ado+EHNA in the presence or absence of pretreatment with 0.01 mM DPCPX for 0.5 h (*n* = 9 per group). Data are mean ± s.e.m. or boxes indicating the interquartile range with whiskers, and dotted linesindicate that significance was calculated separately for each. Significance was determined by one-way ANOVA with Dunnett’s multiple-comparisons (****P < 0.0001) in **a**, **d** and **e** or otherwise by two-way ANOVA with Šidák’s multiple-comparisons test (*P < 0.5, **P < 0.01, ***P < 0.001, ****P < 0.0001). n.s., not significant.

Adenosine receptors are classified into four main families: A1R, A2aR, A2bR and A3R^45–47^. A2bR was predominantly expressed in N41 cells, followed by A1R, while A2aR and A3R showed very low expression levels (Fig. 5a). Therefore, we first targeted *A2br* for knockdown. When *A2br*-knockdown N41 cells were cocultured with *Tspo*-knockdown A2/29 cells in the presence of EHNA, the downregulation of *Agrp* and *Npy* expression by *Tspo* knockdown was not altered (Supplementary Fig. 4e, f). By contrast, *A1r* knockdown abolished the downregulation of *Agrp* and *Npy* expression by *Tspo*-knockdown even when extracellular adenosine levels were highest because of the presence of EHNA and *Tspo-*knockdown (Fig. 5c, Supplementary Fig. 4g). Increases in extracellular ATP and adenosine levels by *Tspo* knockdown were unaffected by *A1r* knockdown (Fig. 5b). Collectively, these results suggest that ATP released from *Tspo-*knockdown A2/29 cells was converted to adenosine, which regulated the expression of *Agrp* and *Npy* via A1R in N41 cells.

Because the increase in extracellular adenosine through the conversion of ATP released from *Tspo*-knockdown A2/29 cells decreased *Agrp* and *Npy* expression in N41 cells, we examined whether direct adenosine treatment of N41 cells would also regulate the mRNA expression of *Agrp* and *Npy*. We used 0.1 mM adenosine because this concentration was nontoxic to N41 cells (Supplementary Fig. 4h), and more importantly, the extracellular adenosine levels in N41 cells treated with 0.1 mM adenosine in the presence of EHNA were similar to those of N41 cells cocultured with *Tspo*-knockdown A2/29 cells under oleic acid treatment in Fig. 4b (Supplementary Fig. 4i). The mRNA expression of both *Agrp* and *Npy* was downregulated by adenosine and EHNA cotreatment (Ado+EHNA) (Fig. 5d, e). Notably, *A1r* knockdown or inhibition of A1R using DPCPX abolished this downregulation (Fig. 5f–i). These data reveal that direct adenosine treatment downregulates the mRNA levels of *Agrp* and *Npy* via A1R in N41 cells.

### Adenosine reduces appetite and *Agrp* and *Npy* expression but not *Pomc* and *Cart* via adenosine A1 receptors in Tan^*Tspo*^ cKO mice

**Fig. 6:**
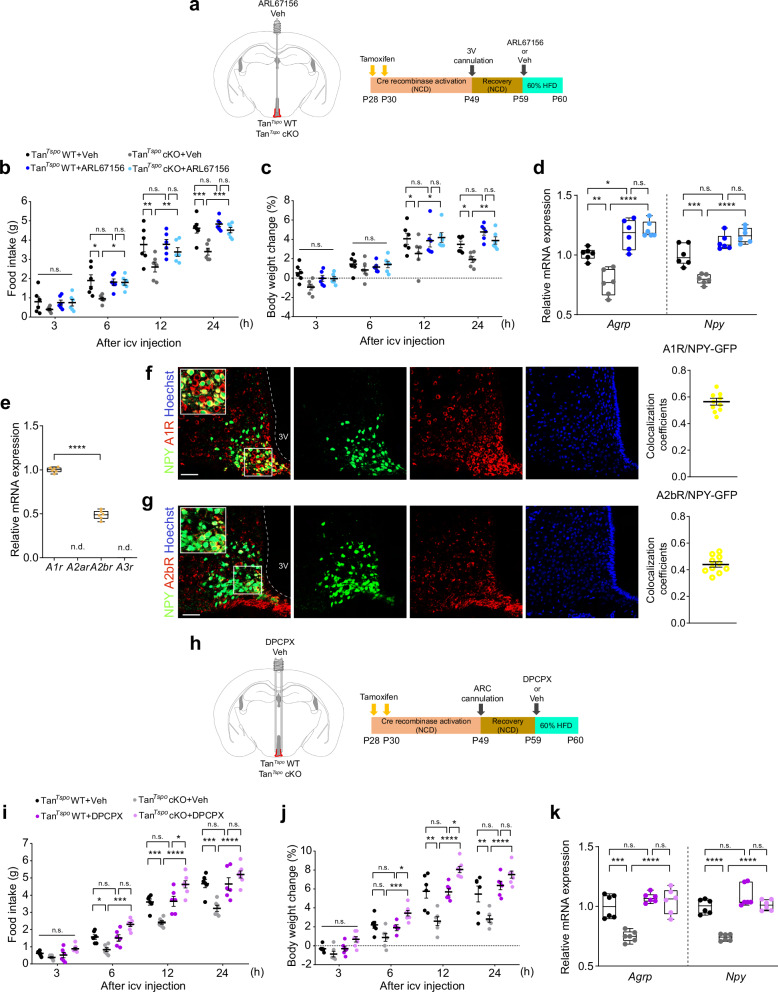
Adenosine converted from extracellular ATP suppresses food intake via adenosine A1 receptor in Tan^*Tspo*^ cKO mice. **a** The schematic strategy and schedule of ARL67156 or Veh ICV injection into the 3V through an implanted cannula in Tan^*Tspo*^ WT or Tan^*Tspo*^ cKO mice. **b**, **c** Food intake (**b**) and body weight change (**c**) of Tan^*Tspo*^ WT and Tan^*Tspo*^ cKO mice for 24 h after ARL67156 (0.1 nmol) or Veh ICV injection and HFD feeding (*n* = 6 per group). **d** Relative *Agrp* and *Npy* expression levels in Tan^*Tspo*^ WT and Tan^*Tspo*^ cKO mice 4 h after ARL67156 or Veh ICV injection and HFD feeding (*n* = 6 per group). **e** Relative expression levels of adenosine receptors in the mouse hypothalamus (*n* = 6). **f** Representative image of A1R (red) expressed on NPY neurons (green) in the hypothalamus and colocalization coefficients of A1R with NPY neurons (*n* = 10). **g** Representative image of A2bR (red) expressed on NPY neurons (green) in the hypothalamus and colocalization coefficients of A2bR with NPY neurons (*n* = 10). **h** The schematic strategy and schedule of A1R inhibitor (DPCPX) or Veh ICV injection into the ARC through an implanted cannula in Tan^*Tspo*^ WT or Tan^*Tspo*^ cKO mice. **i**, **j** Food intake (**i**) and body weight change (**j**) of Tan^*Tspo*^ WT and Tan^*Tspo*^ cKO mice for 24 h after DPCPX (0.4 nmol) or Veh ICV injection and HFD feeding (*n* = 6 per group). **k** Relative *Agrp* and *Npy* expression levels in Tan^*Tspo*^ WT and Tan^*Tspo*^ cKO mice 4 h after DPCPX or Veh ICV injection and HFD feeding (*n* = 6 per group). Scale bars, 20 μm (**f** and **g**). Data are mean ± s.e.m. or boxes indicating the interquartile range with whiskers, and dotted lines indicate that significance was calculated separately for each. Significance was determined by one-way ANOVA with Dunnett’s multiple-comparisons test (****P < 0.0001; n.d., not detected) in **e** or otherwise by two-way ANOVA with Šidák’s multiple-comparisons test (*P < 0.5, **P < 0.01, ***P < 0.001, ****P < 0.0001; n.s., not significant).

To validate that extracellular conversion of ATP in tanycyte-specific *Tspo*-knockout mice regulates the mRNA levels of orexigenic neuropeptides, we administered ARL67156 into the 3V of Tan^*Tspo*^ cKO and Tan^*Tspo*^ WT mice to inhibit ATP conversion (Fig. 6a). In Tan^*Tspo*^ cKO mice, the reductions in food intake and body weight were prevented by ARL67156 injection (Fig. 6b, c). Consistent with the changes observed in N41 cells, the downregulation of *Agrp* and *Npy* expression was also reversed in ARL67156-administered Tan^*Tspo*^ cKO mice (Fig. 6d). ARL67156 had no noticeable effects in Tan^*Tspo*^ WT mice. The expression levels of *Pomc* and *Cart* were not changed in ARL67156-administered Tan^*Tspo*^ cKO mice (Supplementary Fig. 5a).

To investigate the expression of adenosine receptors in AGRP/NPY neurons and POMC/CART neurons in vivo, we analyzed the colocalization of A1R and A2bR in these neurons in NPY-hrGFP and POMC-hrGFP mice, respectively. Notably, A1R was predominant in the whole mouse hypothalamus, particularly in AGRP/NPY neurons rather than POMC/CART neurons (Fig. 6e–g and Supplementary Fig. 5c, d). Given this evidence, we tested whether inhibition of A1R in the hypothalamic ARC regulates food intake, body weight and *Agrp* and *Npy* expression. We measured food intake and body weight during 24 h after injection of dipropylcyclopentylxanthine (DPCPX), a pharmacological inhibitor of A1R, into the hypothalamic ARC of Tan^*Tspo*^ WT and Tan^*Tspo*^ cKO mice (Fig. 6h). In Tan^*Tspo*^ WT mice, DPCPX did not affect food intake and body weight. By contrast, in Tan^*Tspo*^ cKO mice, DPCPX increased food intake and body weight over time compared with vehicle-injected mice (Fig. 6i, j). Relevantly, the mRNA expression of *Agrp* and *Npy* was elevated in DPCPX-injected mice compared with vehicle-injected mice in the Tan^*Tspo*^ cKO group, while that of *Pomc* and *Cart* was unaffected (Fig. 6k and Supplementary Fig. 5b). Taken together, these data suggest a crucial role of A1R in the signaling that regulates the expression of orexigenic neuropeptides in AGRP/NPY neurons.

To further explore whether ATP released from *Tspo*-knockdown A2/29 cells regulated the expression of *Pomc* and *Cart* in a POMC/CART-expressing neuronal cell line, mHypoE-N43/5 (hereafter referred to as N43/5) was cocultured with *Tspo*-knockdown A2/29 cells in the presence of oleic acid. Similar to the *Pomc* and *Cart* expression in HFD-fed Tan^*Tspo*^ cKO mice (Fig. 1f and Supplementary Fig. 2e), the mRNA levels of *Pomc* and *Car*t in N43/5 cells were unaffected by oleic acid (Supplementary Fig. 5e) or Ado+EHNA that inhibit the extracellular conversion of adenosine into inosine by ADA (Supplementary Fig. 5f, g). These results indicate that the mRNA expression of *Pomc* and *Cart* is not modulated by adenosine via A1R.

### Adenosine regulates the expression of *Agrp* and *Npy* by suppressing ERK phosphorylation via adenosine A1 receptors

**Fig. 7:**
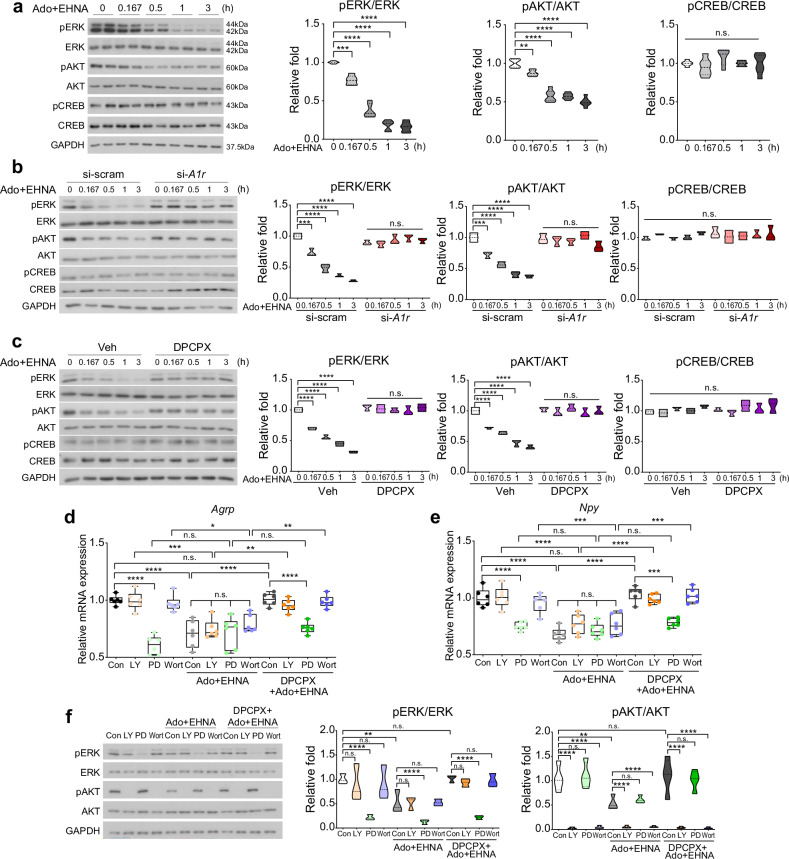
Adenosine A1 receptor-mediated ERK signaling downregulates the expression of *Agrp* and *Npy.* **a**, **b** Western blot analysis of phosphorylation of ERK, AKT and CREB for 3 h after Ado+EHNA in N41 cells (*n* = 6 per group) (**a**) and si-scram or si-*A1r* N41 cells (n = 5 per group) (**b**). **c** Western blot analysis of phosphorylation of ERK, AKT and CREB for 3 h after Ado+EHNA with or without DPCPX in N41 cells (*n* = 5 per group). **d**–**f** Cells were pretreated with a PI3K inhibitor (0.02 mM LY294002, LY; or 0.001 mM wortmannin, Wort) or ERK inhibitor (0.05 mM; PD98059, PD) for 0.5 h before Ado+EHNA treatment in the absence or presence of DPCPX: relative *Agrp* (**d**) and *Npy* (**e**) expression levels in pretreated N41 cells with or without Ado+EHNA and DPCPX treatment for 3 h under LY, PD or Wort treatment (*n* = 6 per group); representative western blots and quantitation of phosphorylation of ERK and AKT in N41 cells after Ado+EHNA treatment for 3 h in the presence or absence of DPCPX treatment under LY, PD, or Wort treatment (n = 6 per group) (**f**). Data are truncated violin plots or boxes indicating the interquartile range with whiskers. Significance was determined by two-way ANOVA with Šidák’s multiple-comparisons test (*P < 0.5, **P < 0.01, ***P < 0.001, ****P < 0.0001) in **d**–**f** or otherwise by one-way ANOVA with Dunnett’s multiple-comparisons test (**P < 0.01, ***P < 0.001, ****P < 0.0001). n.s., not significant; Con; untreated control.

To elucidate the mechanisms by which adenosine regulates the expression of *Agrp* and *Npy* via A1R, we first assessed the signaling downstream of adenosine–A1R. We analyzed the phosphorylation levels of protein kinase B (AKT), extracellular-signal-regulated kinase (ERK) and cAMP response element-binding protein (CREB) in Ado+EHNA-treated N41 cells. Those of ERK and AKT were reduced in a time-dependent manner, while that of CREB remained unchanged throughout the duration of the experiment (Fig. 7a). When *A1r* knockdown or DPCPX treatment was applied to Ado+EHNA-treated N41 cells, there were no decreases in the phosphorylation levels of ERK and AKT, indicating that A1R activation leads to their dephosphorylation (Fig. 7b, c). As the phosphorylation levels of CREB were unaffected under all conditions, the modulation of *Agrp* and *Npy* expression by adenosine signaling via A1R does not involve CREB.

To confirm that ERK and AKT signaling mediates the regulation of *Agrp* and *Npy* expression, we pretreated N41 cells with an ERK inhibitor (PD98059) or PI3K/AKT inhibitors (LY293002 and wortmannin) (Fig. 7d–f). Inhibition of ERK phosphorylation by PD98059 decreased the expression of *Agrp* and *Npy* in comparison with that in untreated cells, whereas inhibition of AKT phosphorylation by LY293002 or wortmannin did not change the expression of these genes, indicating that dephosphorylation of ERK but not AKT is critical in the downregulation of *Agrp* and *Npy* expression (Fig. 7d, e). In line with this result, inhibition of ERK phosphorylation did not further affect the expression levels of *Agrp* and *Npy* reduced by Ado+EHNA. Meanwhile, PD98059 downregulated *Agrp* and *Npy* expression under Ado+EHNA even in the presence of the A1R inhibitor DPCPX, suggesting that ERK dephosphorylation occurs downstream of A1R activation (Fig. 7d, e). The phosphorylation of AKT was significantly reduced upon LY293002 and wortmannin treatment, while ERK phosphorylation was markedly decreased after PD98059 treatment. These inhibitory effects on phosphorylation were consistently observed in the presence of Ado+EHNA or DPCPX+Ado+EHNA (Fig. 7f). Collectively, these data indicate that adenosine signaling through A1R reduces the expression of *Agrp* and *Npy* by inactivating the ERK pathway in N41 cells.

### Inhibition of adenosine A1 receptors in the AGRP/NPY neurons abolishes the effect of adenosine on reducing appetite and neuropeptide expression

**Fig. 8:**
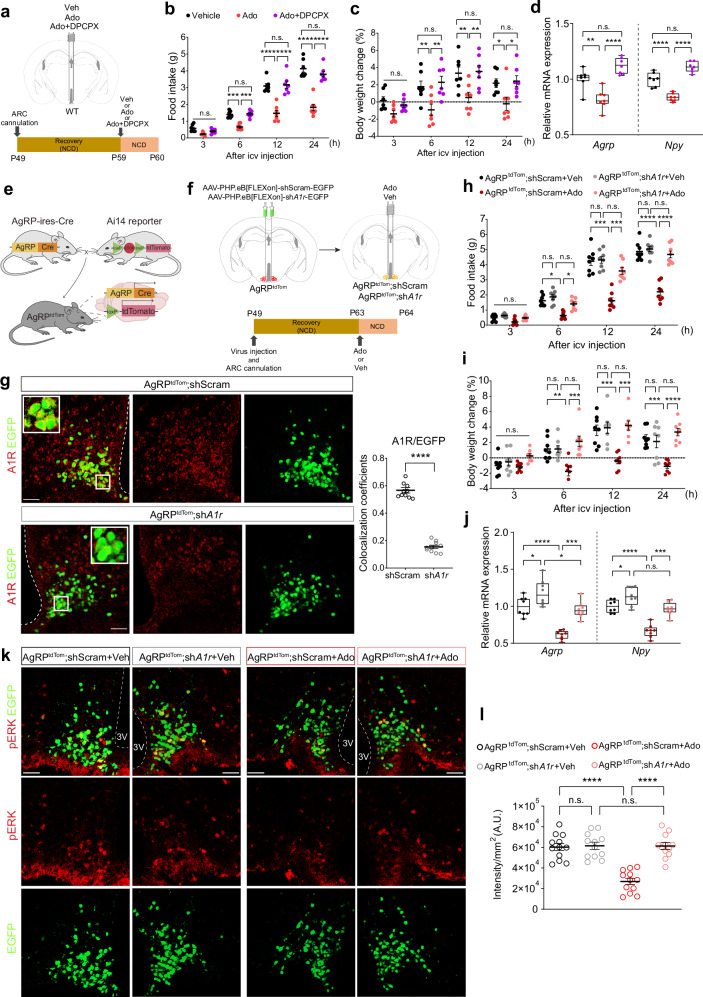
Inhibition of adenosine A1 receptors in the AGRP/NPY neurons abolishes the effect of adenosine on reducing appetite and neuropeptide expression. **a** The schematic schedule of ICV injection of Veh, 10 nmol Ado or 10 nmol Ado + 0.4 nmol DPCPX (Ado+DPCPX) into the ARC through an implanted cannula in WT mice fed NCD. **b**, **c** Food intake (**b**) and body weight change (**c**) of WT mice for 24 h after ICV injection (*n* = 7 per group). **d** Relative *Agrp* and *Npy* expression levels in mice 4 h after ICV injection (*n* = 7 per group). **e** AgRP-ires-cre mice bred with Ai14-tdTomato reporter mice to generate AGRP neuron-specific tdTomato-expressing mice (AgRP^tdTom^). **f** The schematic strategy and schedule of AGRP neuron-specific *A1r* knockdown using AAV injection into the ARC of AgRP^tdTom^ mice and ICV injection of Veh or 10 nmol Ado in AgRP neuron-specific *A1r-*knockdown mice (AgRP^tdTom^;sh*A1r*) or control mice (AgRP^tdTom^;shScram) fed NCD. **g** Representative immunostaining images and colocalization coefficients of A1R (red) with EGFP (green) in AgRP^tdTom^;shScram and AgRP^tdTom^;sh*A1r* mice (*n* = 10). **h**, **i** Food intake (**h**) and body weight change (**i**) of AgRP^tdTom^;shScram and AgRP^tdTom^;sh*A1r* mice for 24 h after Ado or Veh ICV injection (*n* = 8 per group). **j** Relative *Agrp* and *Npy* expression levels in AgRP^tdTom^;shScram and AgRP^tdTom^;sh*A1r* mice 4 h after Ado or Veh ICV injection (*n* = 8 per group). **k** Representative immunostaining images of pERK (red) in AgRP^tdTom^;shScram and AgRP^tdTom^;sh*A1r* mice 4 h after Ado or Veh ICV injection. **l** The fluorescence intensity of pERK colocalized with EGFP-positive cells in AgRP^tdTom^;shScram and AgRP^tdTom^;sh*A1r* mice 4 h after Ado or Veh ICV injection (*n* = 12 per group). Scale bar, 20 μm (**g** and **k**). Data are mean ± s.e.m. or boxes indicating the interquartile range with whiskers, and dotted lines indicate that significance was calculated separately for each. Significance was determined by two-tailed unpaired Student’s *t*-test (****P < 0.0001) in **g** two-way ANOVA with Tukey’s multiple-comparisons test (*P < 0.5, **P < 0.01, ***P < 0.001, ****P < 0.0001) in **b**–**d** or otherwise two-way ANOVA with Šidák’s multiple-comparisons test (*P < 0.5, **P < 0.01, ***P < 0.001, ****P < 0.0001). n.s., not significant.

To investigate whether adenosine reduces appetite via A1R in the hypothalamic ARC, we injected WT mice with adenosine alone or together with DPCPX (Ado+DPCPX) (Fig. 8a). Administration of adenosine alone decreased food intake and body weight after 6 h, whereas Ado+DPCPX abrogated the anorexic effects of adenosine (Fig. 8b, c). In addition, the decrease in *Agrp* and *Npy* mRNA levels by adenosine was reversed by Ado+DPCPX (Fig. 8d).

We generated AGRP/NPY neuron-specific tdTomato-expressing mice (AgRP^tdTom^) by crossing AgRP-ires-cre mice with Ai14-tdTomato reporter mice (Fig. 8e). To assess whether adenosine suppresses appetite via A1R in AGRP/NPY neurons, adenosine or vehicle was injected into the ARC of AgRP neuron–specific *A1r-*knockdown mice (AgRP^tdTom^;sh*A1r*) or control mice (AgRP^tdTom^;shScram) (Fig. 8f). While there was no difference in virus transduction efficiency between shScram and sh*A1r*, the expression of A1R in AGRP neurons was successfully knocked down (Fig. 8g and Supplementary Fig. 6a). Importantly, the reducing effects of adenosine on food intake and body weight were completely abolished by silencing A1R in AGRP/NPY neurons (Fig. 8h, i), consistent with the reversing effects of adenosine on the mRNA levels of *Agrp* and *Npy* in the hypothalamus of AgRP^tdTom^;sh*A1r* mice (Fig. 8j). Collectively, these data indicate that adenosine reduces appetite via A1R by downregulating the expression of orexigenic neuropeptides in AGRP/NPY neurons.

To investigate whether ERK phosphorylation mediates adenosine–A1R signaling in AGRP/NPY neurons, adenosine was injected into the hypothalamic ARC of AgRP^tdTom^;sh*A1r* mice. In contrast to the decrease in ERK phosphorylation in adenosine-injected AgRP^tdTom^;shScram mice, the phosphorylation levels of ERK remained unchanged in AgRP^tdTom^;sh*A1r* mice upon adenosine injection (Fig. 8k, l and Supplementary Fig. 6b). Thus, consistent with the results obtained in N41 cells, adenosine reduced *Agrp* and *Npy* expression by suppressing ERK phosphorylation via A1R in AGRP/NPY neurons.

## Discussion

The hypothalamic neuron–glia relay circuit may play a critical role in harmonic control of energy homeostasis^3–5,8^. Our understanding of how tanycytes perform their tasks as a component of hypothalamic regulatory networks is still in the beginning stages. Our study provides compelling evidence that tanycytes communicate with AGRP/NPY neurons in the hypothalamic ARC to regulate the expression of neuropeptides critical for controlling food consumption and body weight via adenosine signaling in Tan^*Tspo*^ cKO mice.

In contrast to prior investigations that primarily relied on ATP biosensors in primary tanycyte cultures or ex vivo brain slices to detect ATP release^41,42,48^, our study adopted an in vivo approach to monitor ATP release within the hypothalamus using luciferase expressed on the cell surface^39,40^. Measuring ATP release in vivo not only allows direct real-time observations under physiological conditions but also provides an understanding of ATP dynamics within the hypothalamus. Cx43s are involved in the tanycytic network by forming gap junctions for intercellular communication of tanycytes^49,50^. However, Cx43 hemichannels allow the trafficking of chemicals between the cytoplasm and the extracellular environment, including ATP release in tanycytes^41,51^. Aligned with the previous study^41^, our results with Gap26 administration demonstrate that Cx43 hemichannels mediate ATP release in Tan^*Tspo*^ cKO-LUC mice.

ATP is released from tanycytes in response to stimuli such as glucose and amino acids^41,42,48^. However, how the released ATP works and what its role is have not been clearly elucidated. Our findings provide new insights into the role of adenosine converted from released ATP as a gliotransmitter in the extracellular space of tanycytes in regulating orexigenic neuropeptide expression in AGRP/NPY neurons. Extracellular ATP is converted to ADP, AMP and adenosine by ectonucleotidase families in immune and glial cells^52,53^. ENTPD subtype 2 is the ectonucleotidase expressed in tanycytes^54^. Indeed, these ectonucleotidases converted ATP to adenosine in tanycyte-like cells, leading to changes in extracellular ATP and adenosine levels. Although adenosine is converted to inosine by ADA and increased extracellular adenosine is transported into tanycytes via ENT1, the extracellular adenosine levels in *Tspo*-knockdown cells were maintained high enough to downregulate *Agrp* and *Npy* mRNA expression in N41 cells. Consistently, the release of ATP and its conversion to adenosine in Tan^*Tspo*^ cKO mice suppressed food intake by downregulating *Agrp* and *Npy* expression in AGRP/NPY neurons. Therefore, in Tan^*Tspo*^ cKO mice and *Tspo*-knockdown tanycyte cells, adenosine converted from the released ATP, but not ATP as such, acts as an anorexigenic gliotransmitter.

Among adenosine receptors, mainly A1R is expressed in ARC neurons^55,56^. However, knowledge about the cellular sources of signals received by purinergic receptors on ARC neurons and downstream signaling of these receptors is still lacking. Our result shows that A1R is located on AGRP/NPY neurons rather than POMC/CART neurons. A1R expressed on oxytocin neurons in the paraventricular nucleus of the hypothalamus is inhibited by caffeine to increase energy expenditure and reduce food intake in DIO mice^56^. Furthermore, chemogenetic stimulation of astrocytes inhibited AGRP neuron activity via the A1R signaling pathway, reducing food intake^36^. This suggests that A1R in ARC neurons is important in mediating the interaction between astrocytes and neurons. We explored whether adenosine signaling via A1R mediates the interaction between tanycytes and neurons. A1R activation induces phosphorylation of ERK1/2, promotes K^+^ efflux and inhibits Ca^2+^ influx in ovary cells, embryonic kidney cells and cardiomyoblasts^47,57–60^. In our study, adenosine acted on A1R in N41 cells, decreasing ERK phosphorylation and suppressing *Agrp* and *Npy* expression. Therefore, hypophagia and ERK dephosphorylation in AGRP/NPY neurons observed in AgRP^tdTom^;shScram mice injected with adenosine were prevented in AgRP^tdTom^;sh*A1r* mice even after adenosine injection. These findings suggest that adenosine signaling through A1R in AGRP/NPY neurons inhibits ERK phosphorylation and restrains the expression of *Agrp* and *Npy*, thereby suppressing appetite. By contrast, A1R and A2bR expression levels in both POMC-hrGFP mice and hypothalamic POMC/CART-expressing cells were very low, explaining no discernible effect of adenosine on *Pomc* and *Cart* expression. Although adenosine from tanycytes did not play a role in communication with POMC neurons in our study, it was reported that the interaction between tanycytes and POMC neurons was important for glucose homeostasis^50^. Tanycytes convert glucose to lactate, which is delivered to POMC neurons through monocarboxylate transporters (MCTs) 1/4 and 2 in tanycytes and POMC neurons, respectively. Unlike the Cx43 hemichannels that transmit and release ATP for adenosine signaling to AGRP/NPY neurons in our study, the extensive formation of the Cx43 gap junction is required for lactate transmission to POMC neurons. Interestingly, the food intake of mice lacking tanycytic *Mct1/4* was comparable to the WT despite altered meal patterns and weight gain. Therefore, the tanycytic intercellular communication with AGRP/NPY or POMC neurons for regulating energy homeostasis could be distinctive with different physiological outcomes.

A previous study reported that optogenetic activation of tanycytes by Ca^2+^-permeable channelrhodopsin induces ATP release, leading to the depolarization of ARC neurons ex vivo and a transient increase in food intake in vivo^61^. Unlike optogenetic stimulation of tanycytes, which indiscriminately activates signaling pathways through nonlocalized Ca^2+^ influx, our genetic deletion of *Tspo* in tanycytes activated selective pathways that impact feeding behavior. ATP is the agonist of purinergic receptors, including P2XR and P2YR families^62^. We confirmed that inhibition of ATP conversion by ARL67156 triggers CamKII phosphorylation and subsequently CREB phosphorylation, which upregulates *Agrp* and *Npy* expression in N41 cells via P2X4R (data not shown). Also, administration of the DPCPX, an A1R inhibitor, leads to a slight increase in food intake and body weight in Tan^*Tspo*^ cKO mice compared with Tan^*Tspo*^ WT mice, suggesting that ATP may mediate these effects when adenosine signaling is blocked. By contrast, adenosine converted from ATP has an inverse effect, suggesting opposite roles for ATP and adenosine in regulating *Agrp* and *Npy* expression. In physiological conditions, where the conversion of ATP released from tanycytes in Tan^*Tspo*^ cKO mice is not pharmacologically inhibited, the generated adenosine acts as an anorexigenic gliotransmitter, downregulating the expression of *Agrp* and *Npy* neuropeptides by suppressing ERK downstream of A1R.

A limitation of this study is that confirmation of the main findings using primary tanycytes or cell-sorted tanycytes is needed because A2/29 cells do not perfectly replicate all aspects of tanycytes. Despite this point, our findings on the role of tanycytic adenosine transmission in A2/29 cells may help to improve our understanding of the extended function of tanycytic TSPO in communication between tanycytes and AGRP/NPY neurons.

In conclusion, our works reveal a previously unexplored interaction between tanycytes and AGRP/NPY neurons for regulating appetite and body weight, mediated by adenosine signaling via A1R. Adenosine as a gliotransmitter downregulates orexigenic neuropeptides, linking a newly discovered role in transmitting metabolic signals from tanycytes to neurons and regulating energy homeostasis. Our findings contribute to understanding the intricate mechanisms governing metabolic regulation via communication between tanycytes and AGRP/NPY neurons in the hypothalamus, which may have potential therapeutic implications for obesity and diabetes.

## Supplementary information

Supplementary Information
