# Supplementary material for: Adenosine transmission from hypothalamic tanycytes to AGRP/NPY neurons regulates energy homeostasis
Source: Exp Mol Med. 2025 May 2;57(5):1047–63. doi: 10.1038/s12276-025-01449-6 (PMC12130337; doi:10.1038/s12276-025-01449-6)
Supplement: Supplementary file 1 — Supplementary Information [file 12276_2025_1449_MOESM1_ESM.pdf]

Supplementary Figure 1

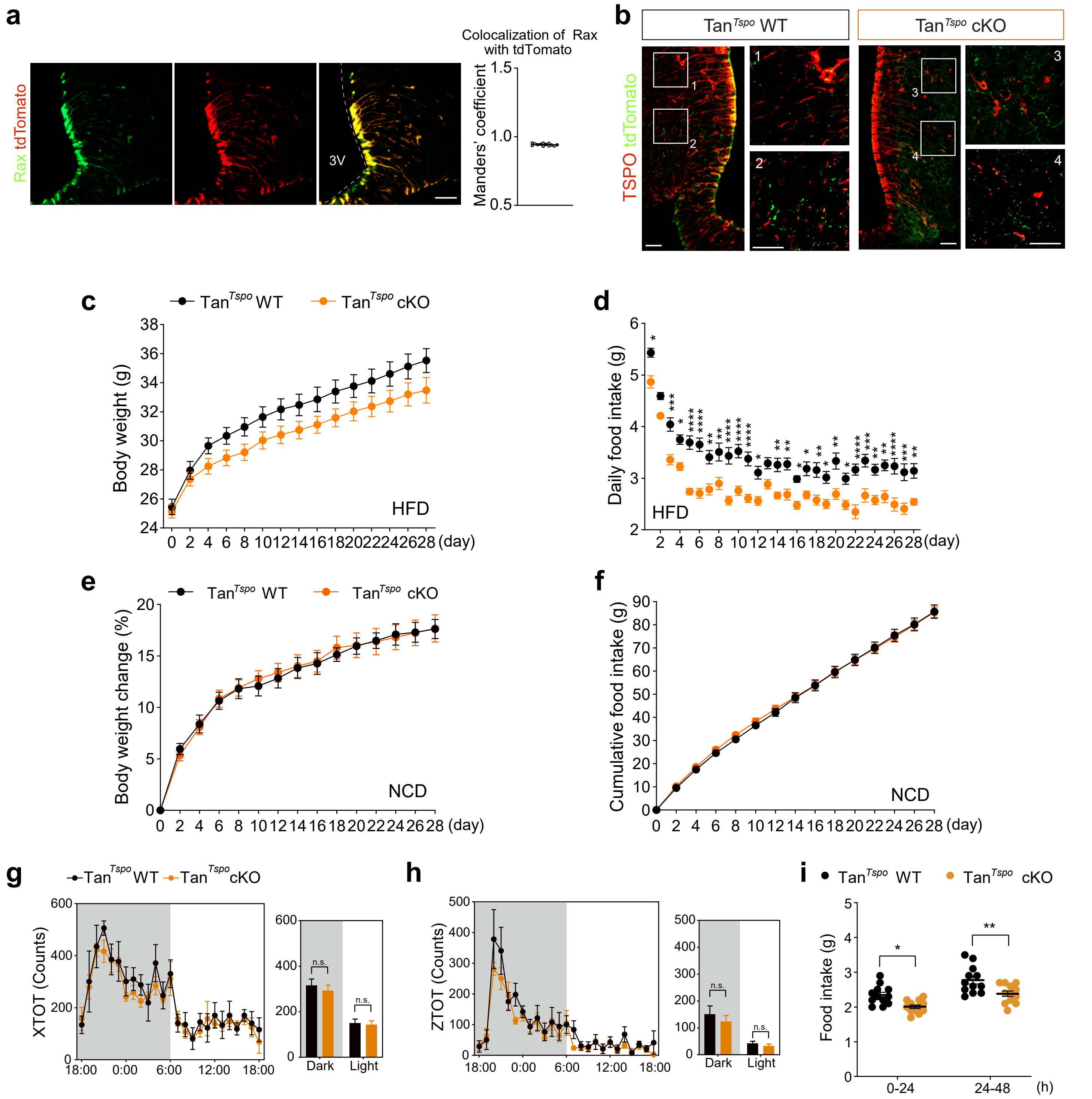

Supplementary Figure 1. Additional phenotypes of Tan^{Tspo} cKO versus Tan^{Tspo} WT male mice. Related to Figure 1. **a** Representative image and quantification of Rax (green) colocalized with tdTomato (red) in Tan^{Tspo} WT mice. **b** Representative images of TSPO (green) colocalized with tdTomato (red) in Tan^{Tspo} WT and Tan^{Tspo} cKO mice. White boxes indicated the mediobasal hypothalamus. **c** Body weight (g) and **d** daily food intake in Tan^{Tspo} WT and Tan^{Tspo} cKO male mice during 4 weeks of high-fat diet (HFD) (n=12 per group). **e** Body weight change (%) and **f** cumulative food intake during 4 weeks of normal chow diet (NCD) in Tan^{Tspo} WT and Tan^{Tspo} cKO male mice (n=5 per group). Locomotor activities of **g** *X*-axis and **h** *Z*-axis in Tan^{Tspo} WT and Tan^{Tspo} cKO male mice after 4 weeks of HFD. **i** Daily food intake during indirect calorimetry measurements in Tan^{Tspo} WT and Tan^{Tspo} cKO male mice fed HFD. Scale bar in **a** and **b**, 20 μm. Data represent mean±s.e.m. Significance was determined by two-way ANOVA with Sidak's multiple comparisons test (*p<0.05, **p<0.01, ***p<0.001, ****p<0.0001; n.s., not significant).

Supplementary Figure 2

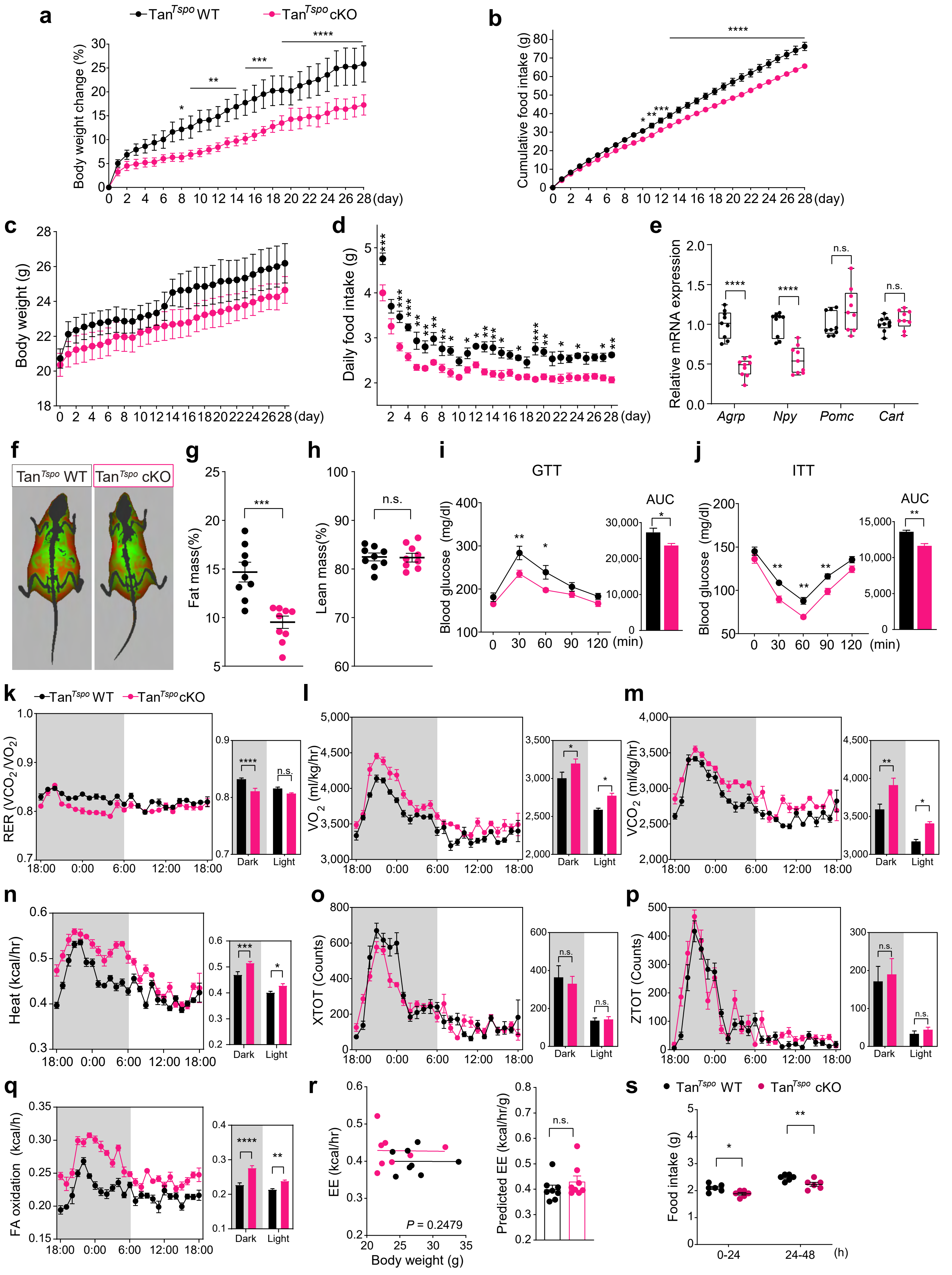

Supplementary Figure 2. Tan^{Tspo} cKO female DIO mice show a reduction of appetite and improvement of glucose homeostasis. a Body weight change (%), **b** cumulative food intake, **c** body weight (g), and **d** daily food intake during 4 weeks of HFD in Tan^{Tspo} WT and Tan^{Tspo} cKO female mice (n=9 per group). **e** Relative mRNA levels of neuropeptides in Tan^{Tspo} WT and Tan^{Tspo} cKO female mice after 4 weeks of HFD (n=9 per group). **f** Representative images of body composition. Red, fat mass; green, lean mass. **g** Fat mass and **h** lean mass after 4 weeks of HFD (n=9 per group). **i** GTT and **j** ITT in Tan^{Tspo} WT and Tan^{Tspo} cKO female mice (n=9 per group). **k–s** Indirect calorimetry parameters in Tan^{Tspo} WT and Tan^{Tspo} cKO female mice after 4 weeks of HFD (n=6 per group); **k** Respiratory exchange ratio (RER), **l** O_2 consumption, **m** CO_2 production, **n** heat generation, **o** and **p** locomotor activities *X*-axis and *Z*-axis, respectively. **q** Fatty acid (FA) oxidation, **r** regression plots of energy expenditure (EE) against body weight and predicted EE at the mean of body weight of each group, and **s** daily food intake during indirect calorimetry measurements. Data represent mean±s.e.m. or boxes indicating the interquartile range with whiskers. Significance was determined by two-tailed unpaired Student's *t*-test (*p<0.05, **p<0.01, ***p<0.001) in **g** and **h**, AUC in **i** and **j**, and using ANCOVA in **r** or otherwise by two-way ANOVA with Sidak's multiple comparisons test (*p<0.05, **p<0.01, ***p<0.001, ****p<0.0001). n.s., not significant; AUC, area under the curve.

Supplementary Figure 3

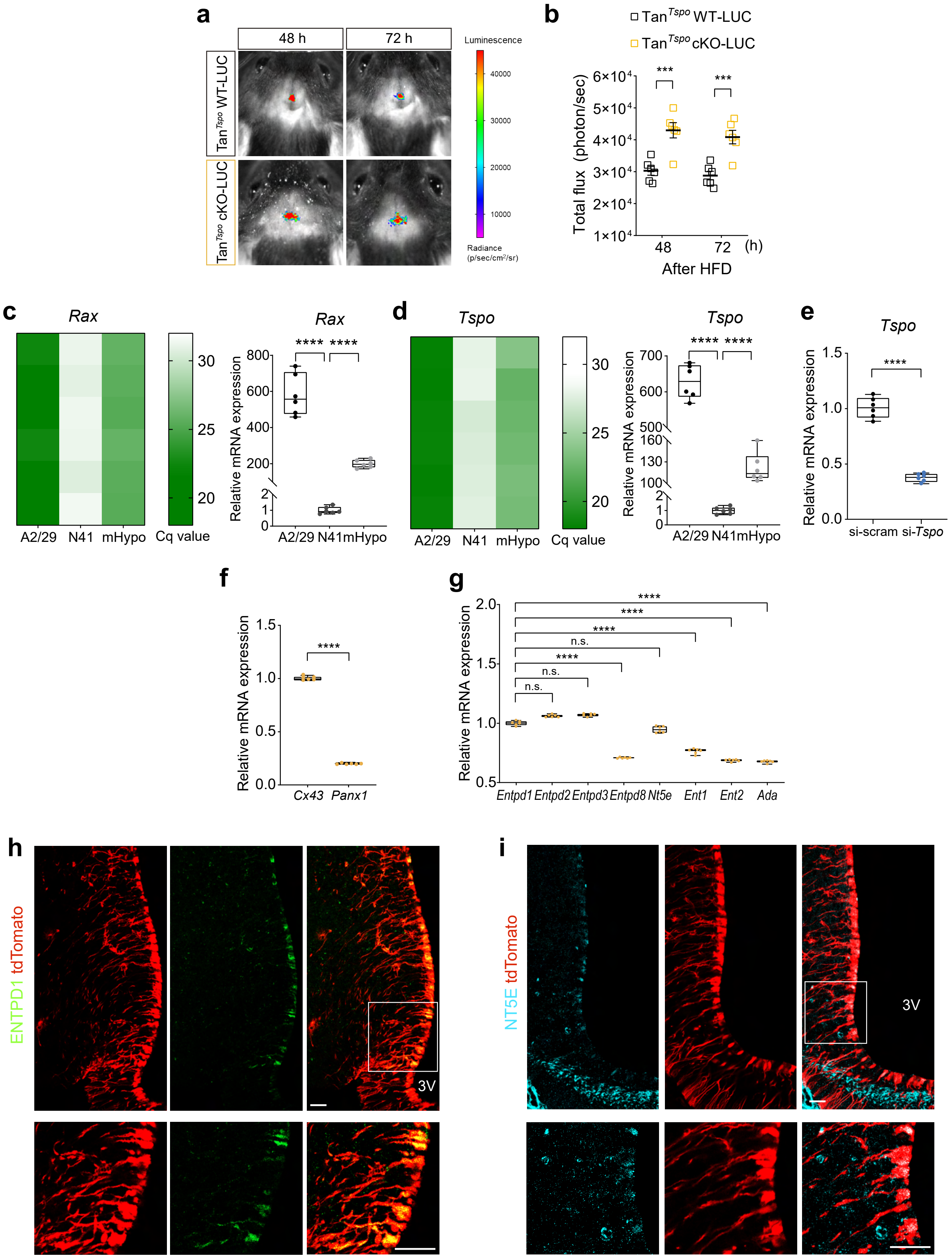

Supplementary Figure 3. Bioluminescence imaging of Tan^{Tspo} WT and Tan^{Tspo} cKO mice and expression of hemichannels, ectonucleotidases, and nucleoside transporters in the hypothalamus. **a–f** Related to Figure 2; **g–i** related to Figure 3. **a** Representative bioluminescence images and **b** quantification of the total flux in Tan^{Tspo} WT and Tan^{Tspo} cKO mice after 48 h and 72 h of HFD feeding (n=6 per group). **c** Heatmap of *Rax* quantification in A2/29 cells, N41 cells, and mouse hypothalamus (left) and relative mRNA expression levels of *Rax* in A2/29 cells, N41 cells, and mouse hypothalamus (right). **d** Heatmap of *Tspo* quantification in A2/29 cells, N41 cells, and mouse hypothalamus (left) and relative mRNA expression levels of *Tspo* in A2/29 cells, N41 cells, and mouse hypothalamus (right). **e** Efficiency of knockdown using si-*Tspo* in A2/29 cells (n=6 per group). Relative mRNA expression of **f** *Cx43* and *Panx1* and **g** ectonucleotidases and nucleoside transporters in the mouse hypothalamus (n=6). Representative immunostaining images of **h** ENTPD1 (green) and **i** NT5E (turquoise) in tanycytes (red). Lower images are enlargements of the white boxes in the upper images. Scale bars in **h** and **i**, 20 μm. Data are mean±s.e.m. or boxes indicating the interquartile range with whiskers. Significance was determined by two-way ANOVA with Sidak's multiple comparisons test (***p<0.001, ****p<0.0001) in **b**, two-way ANOVA with Tukey's multiple comparisons test (****p<0.0001) in **c** and **d**, two-tailed unpaired Student's *t*-test (****p<0.0001) in **e** and **f**, and one-way ANOVA with Dunnett's multiple comparisons test (****p<0.0001) in **g**. mHypo, mouse hypothalamus; n.s., not significant.

Supplementary Figure 4

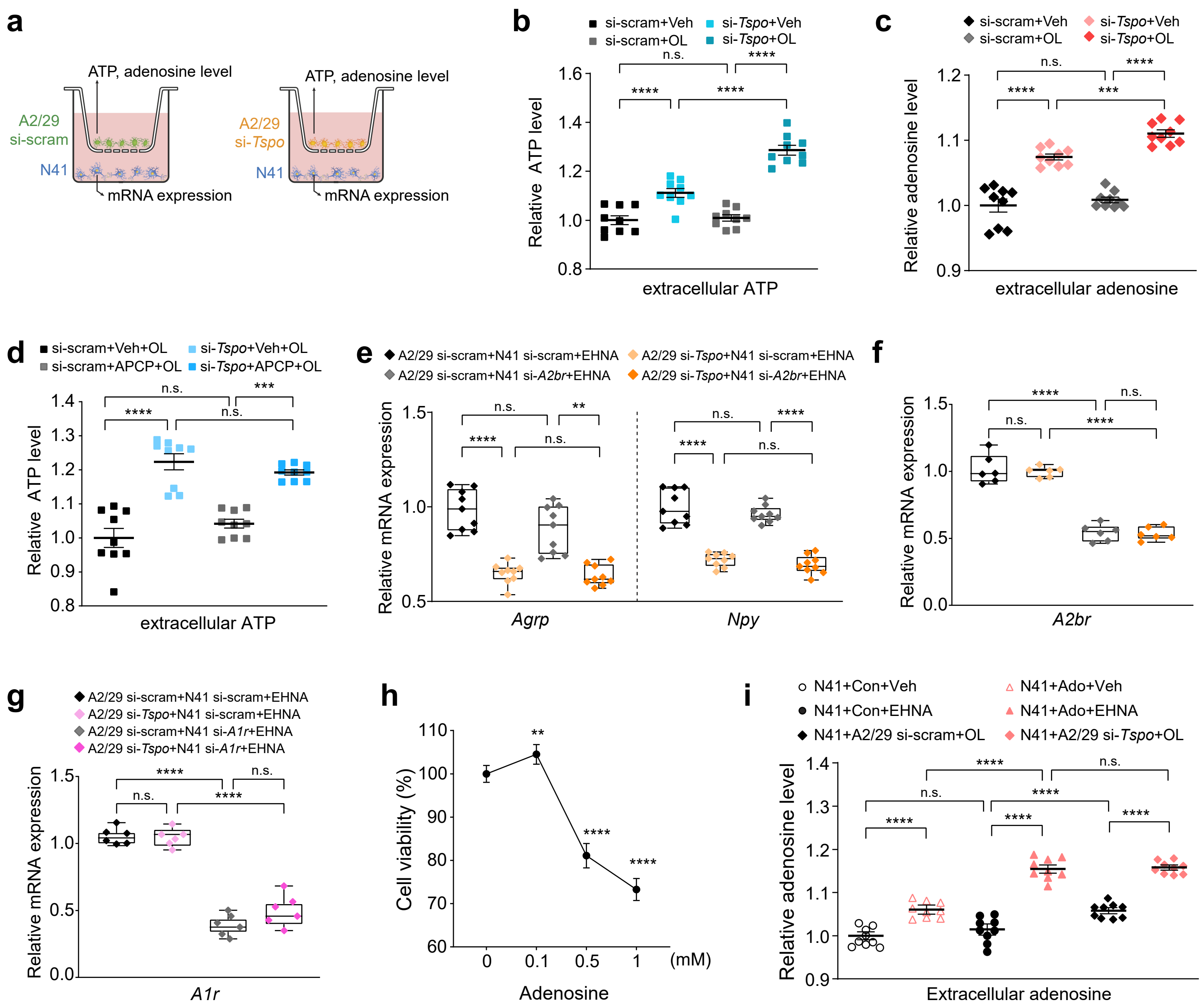

Supplementary Figure 4. Expression of orexigenic neuropeptides in co-culture systems with A2/29 and N41 cells and optimization of the concentration of adenosine. a–d Related to Figure 4; **e–i** related to Figure 5. **a** Schematic illustration of indirect co-culture using a transwell to investigate intercellular crosstalk between A2/29 cells and hypothalamic AGRP/NPY-expressing cells (mHypoE-N41, N41). A2/29 cells were grown on permeable membranes, transfected with si-RNAs, and then transferred over the wells containing N41 cells, which were separately cultured at the bottom of the wells. A2/29 cells were collected to examine the ATP and adenosine levels and N41 cells were used for analysis of mRNA expression. **b** Relative extracellular ATP levels and **c** relative extracellular adenosine levels in si-scram or si-*Tspo* A2/29 cells with or without OL treatment for 3 h (n=9 per group). **d** Relative extracellular ATP levels in si-scram or si-*Tspo* A2/29 cells indirectly co-cultured with N41 cells in the presence or absence of APCP under OL treatment for 3 h (n=9 per group). **e** Relative *Agrp* and *Npy* expression levels in si-scram or si-*A2br* N41 cells indirectly co-cultured with si-scram or si-*Tspo* A2/29 cells for 3 h under EHNA treatment (n=9 per group). Efficiency of knockdown using **f** si-*A2br* or **g** si-*A1r* in N41 cells (n=6 per group). **h** Concentration-dependent viability of N41 cells for 24 h (n=6). **i** Extracellular adenosine levels in N41 cells in the presence or absence of EHNA with or without adenosine (Ado) treatment and in N41 cells co-cultured with *Tspo*-knockdown A2/29 cells under OL treatment for 3 h (n=9 per group). Data are mean±s.e.m. or boxes indicating the interquartile range with whiskers and dot lines separate individual significance. Significance was determined by one-way ANOVA with Dunnett's multiple comparisons test (**p<0.01, ****p<0.0001) in **h** or otherwise by two-way ANOVA with Sidak's multiple comparisons test (*p<0.05, **p<0.01, ***p<0.001, ****p<0.0001). n.s., not significant; Con, untreated control.

Supplementary Figure 5

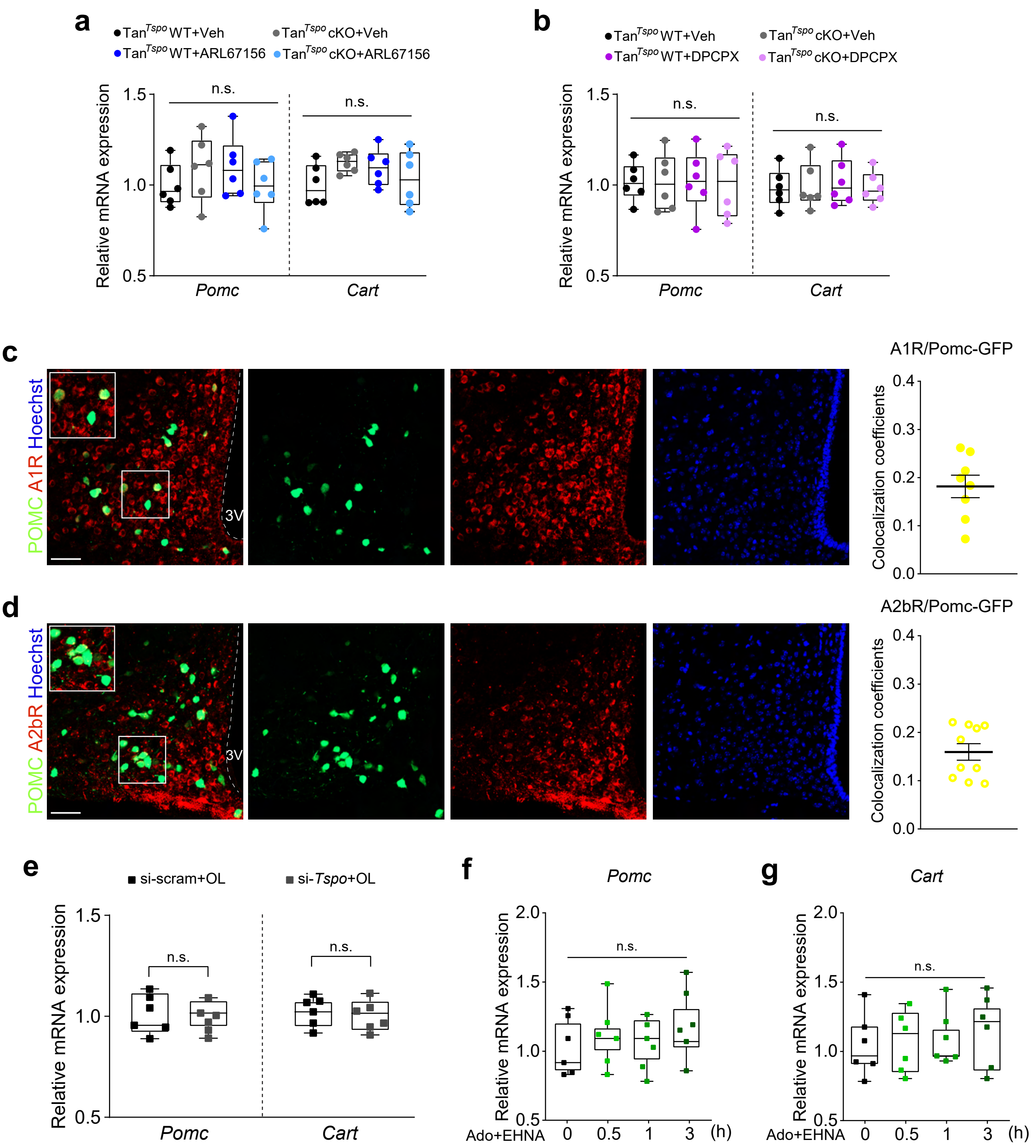

Supplementary Figure 5. Adenosine does not affect *Pomc* and *Cart* expression via adenosine A1 receptors. Related to Figure 6. **a** Relative mRNA levels of *Pomc* and *Cart* in Tan^{Tspo} WT and Tan^{Tspo} cKO mice injected with Veh or ARL67156 4 h after icv injection and HFD feeding (n=6 per group). **b** Relative mRNA levels of *Pomc* and *Cart* in Tan^{Tspo} WT and Tan^{Tspo} cKO mice injected with Veh or DPCPX 4 h after icv injection and HFD feeding (n=6 per group). **c** Representative image of A1R (red) expressed in POMC neurons (green) in the hypothalamus and colocalization coefficients of A1R with POMC neurons (n=8). **d** Representative image of A2bR (red) expressed at POMC neurons (green) in the hypothalamus and colocalization coefficients of A2bR with POMC neurons (n=10). **e** Relative mRNA levels of *Pomc* and *Cart* in hypothalamic POMC/CART-expressing cells (mHypoE-N43/5, N43/5) indirectly co-cultured with si-scram or si-*Tspo* A2/29 cells under 0.06 mM OL treatment for 3 h (n=6 per group). Time course of relative **f** *Pomc* and **g** *Cart* expression levels in N43/5 cells treated with Ado+EHNA (n=6 per group). Scale bars in **c** and **d**, 20 μm. Data are mean±s.e.m. or boxes indicating the interquartile range with whiskers and dot lines separate individual significance. Significance was determined by two-way ANOVA with Sidak's multiple comparisons test in **a** and **b**, two-tailed unpaired Student's *t*-test in **e**, and by one-way ANOVA with Dunnett's multiple comparisons test in **f** and **g**. n.s., not significant.

Supplementary Figure 6

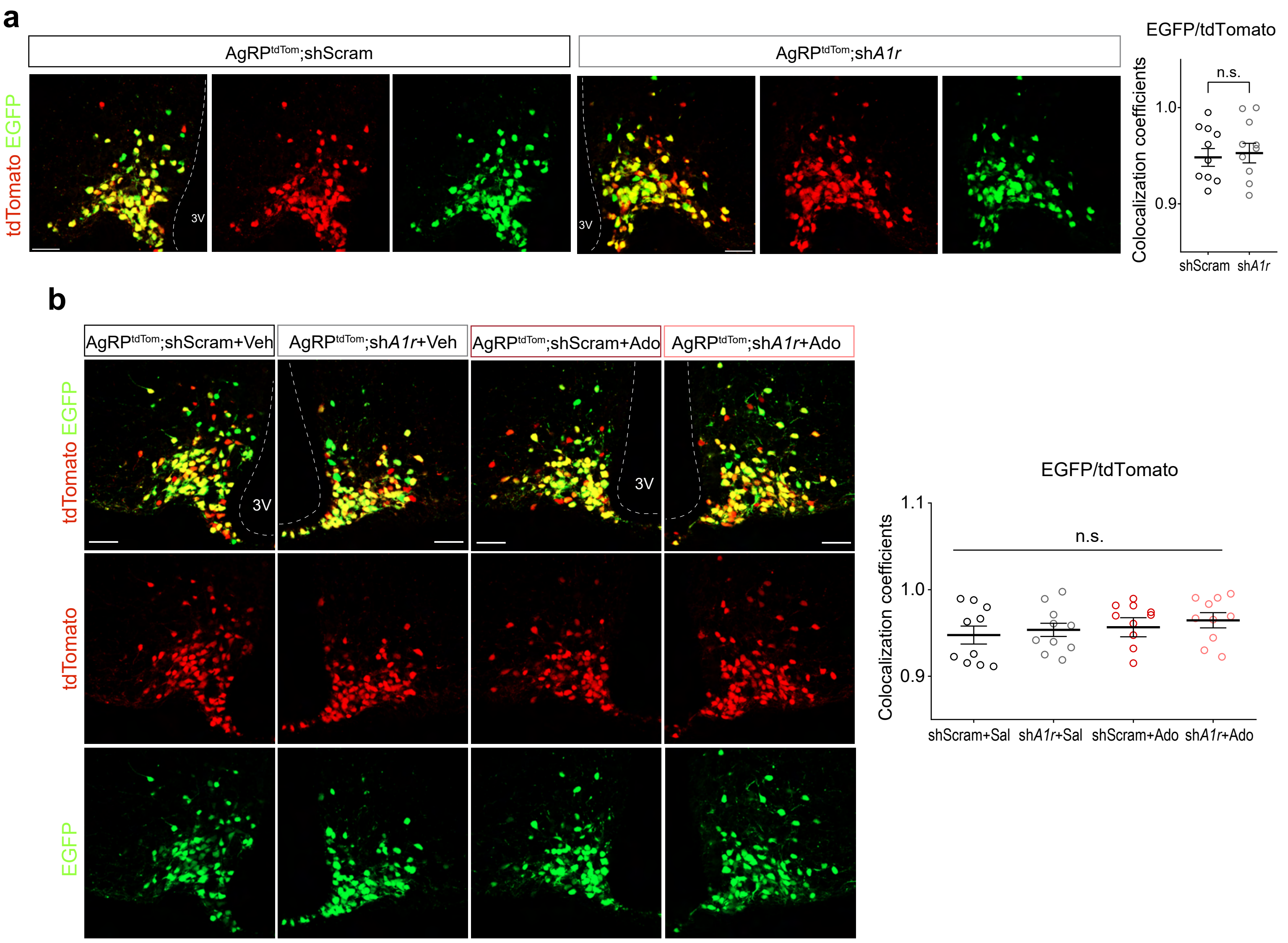

Supplementary Figure 6. The efficiency of AAV transduction in AgRP^{tdTom};shScram and AgRP^{tdTom};Sh*A1r*. Related to Figure 8. **a** Representative images and analysis of the functional efficiency of AAV in AgRP^{tdTom};shScram and AgRP^{tdTom};sh*A1r* mice (n=10 per group). **b** Representative images and colocalization coefficients of tdTomato (red) and EGFP (green) in AgRP^{tdTom};shScram and AgRP^{tdTom};sh*A1r* mice with or without Ado icv injection (n=10 per group). Scale bar, 20 μm. Data are mean±s.e.m. Significance was determined by two-tailed unpaired Student's *t*-test in **a** and by two-way ANOVA with Sidak's multiple comparisons test in **b**. n.s., not significant.

Supplementary Table 1. The sequence of primers for genotyping PCR

Primer	Sequence (5′ to 3′)
$Tspo^{fl/fl}$ forward	GGA TTA CCA CAC CCA ACC AG
$Tspo^{fl/fl}$ reverse	TAG GAG TGC AAA GCC AGT CA
Rax-Cre^{ERT2} forward-common	CCC TGA GGC TAA ACT TGC AG
Rax-Cre^{ERT2} reverse-wild	AGG TGT CTA GGA TGC CGT CT
Rax-Cre^{ERT2} reverse-mutant	AGG CAA ATT TTG GTG TAC GG
Ai14-tdTomato forward-wild	AAG GGA GCT GCA GTG GAG TA
Ai14-tdTomato reverse-wild	CCG AAA ATC TGT GGG AAG TC
Ai14-tdTomato forward-mutant	GGC ATT AAA GCA GCG TAT CC
Ai14-tdTomoato reverse-mutant	CTG TTC CTG TAC GGC ATG G
AgRP-ires-Cre	GGG CCC TAA GTT GAG TTT TCC T
AgRP-ires-Cre	GAT TAC CCA ACC TGG GCA GAA C
AgRP-ires-Cre	GGG TCG CTA CAG ACG TTG TTT G

Supplementary Table 2. List of antibodies

Antibodies	Manufacturer
Phospho-p44/42 MAPK (ERK1/2) (Thr202/Tyr204)	Cell Signaling Technology (#4370, #9101)
P44/42 MAPK (ERK1/2)	Cell Signaling Technology (#9107)
Phospho-AKT (Ser473)	Cell Signaling Technology (#4060)
AKT	Cell Signaling Technology (#9272)
Phospho-CREB (Ser133)	Cell Signaling Technology (#9198)
CREB	Cell Signaling Technology (#9197)
GAPDH	Cell Signaling Technology (#2118)
TSPO	Abcam (ab109497)
A1R	Invitrogen (MA5-38140)
A2bR	Abcam (ab229671)
Rax	Takara (M229)
Cx43	Sigma-Aldrich (C6219)
ENTPD1	Cell Signaling Technology (#14481)
NT5E	Cell Signaling Technology (#13160)
Alexa flour® 488 Donkey anti-chicken IgG (H+L)	Jackson ImmunoResearch (703-545-155)
Alexa flour® 647 Donkey anti-chicken IgY(IgG) (H+L)	Jackson ImmunoResearch (703-605-155)
Alexa flour® 488 Donkey anti-mouse IgG (H+L)	Jackson ImmunoResearch (715-545-150)
Alexa flour® 647 Donkey anti-mouse IgG (H+L)	Jackson ImmunoResearch (715-605-151)
Alexa flour® 488 Donkey anti-sheep IgG (H+L)	Jackson ImmunoResearch (713-545-003)
Alexa flour® 647 Donkey anti-sheep IgG (H+L)	Jackson ImmunoResearch (713-605-147)
Alexa flour® 488 Donkey anti-rabbit IgG (H+L)	Jackson ImmunoResearch (711-545-152)
Alexa flour® 647 Donkey anti-rabbit IgG (H+L)	Jackson ImmunoResearch (711-605-152)
Alexa flour® 488 Donkey anti-guinea pig IgG (H+L)	Jackson ImmunoResearch (706-545-148)

Supplementary Table 3. The sequence of primers for qRT-PCR

Gene	Forward primer sequence (5′ to 3′)	Reverse primer sequence (5′ to 3′)
Cx43	TCA TCT TCA TGC TGG TGG TGT CCT	TGG TGA GGA GCA GCC ATT GAA GTA
Panx1	GCT CCC TGC AGA GCG AGT CTG G	CTC TTG GCA GCC TTG ATG GCG C
Entpd1	AGC TGC CCC TTA TGG AAG AT	TCA GTC CCA CAG CAA TCA AA
Entpd2	TTC CTG GGA TGT CAG GTC TC	GTC TCT GGT GCT TGC CTT TC
Entpd3	ACC TGT CCC GTG CTT AAA TG	AGA CAG AGT GAA GCC CCT GA
Entpd8	CAC ACA GGA CCT TCT GAG CA	AGC CTT CTG AGG TGG CAC TA
Nt5e	CAG GAA ATC CAC CTT CCA AA	AAC CTT CAG GTA GCC CAG GT
Ent1	CTT GGG ATT CAG GGT CAG AA	ATC AGG TCA CAC GAC ACC AA
Ent2	CAT GGA AAC TGA GGG GAA GA	GTT CCA AAG GCC TCA CAG AG
Ada	ACA CCC GCA TTC AAC AAA CC	TGC CTC TCT TCT TGC CAA AGT
Agrp	CTG CAG ACC GAG CAG AAG A	TGC GAC TAC AGA GGT TCG TG
Npy	CAG AAA ACG CCC CCA GAA	AAA AGT CGG GAG AAC AAG TTT CAT T
Gapdh	ATC ACT GCC ACC CAG AAG AC	ACA CAT TGG GGG TAG GAA CA
A1r	GCA AGA GGC GGA CAT CAC A	TCA CTC ACC CTA GAA GCC ATA CTC
A2ar	CTT TGT CCT GGT CCT CAC GC	TCG CAA TGA TGC CCT TCG
A2br	TCT TCC TCG CCT GCT TCG T	CTC GTG TCC CAG TGA CCA AAC
A3r	CGG GAG TTC AAG ACA GCT AAG T	CAC ATT GCG ACA TCT GGT ATC T
Tspo	TGC AGA AAC CCT CTT GGC ATC	GAA ACC TCC CAG CTC TTT CC
Pomc	GAA CAG CCC CTG ACT GAA AA	ACG TTG GGG TAC ACC TTC AC
Cart	CGA GAA GAA GTA CGG CCA AGT CC	GGA ATA TGG GAA CCG AAG GTG G
